# Factors associated with sharing e-mail information and mental health survey participation in large population cohorts

**DOI:** 10.1093/ije/dyz134

**Published:** 2019-07-01

**Authors:** Mark J Adams, W David Hill, David M Howard, Hassan S Dashti, Katrina A S Davis, Archie Campbell, Toni-Kim Clarke, Ian J Deary, Caroline Hayward, David Porteous, Matthew Hotopf, Andrew M McIntosh

**Affiliations:** 1 Division of Psychiatry, Royal Edinburgh Hospital, Edinburgh, UK; 2 Centre for Cognitive Ageing and Cognitive Epidemiology, Edinburgh, UK; 3 Department of Psychology, University of Edinburgh, Edinburgh, UK; 4 Institute of Psychiatry, Psychology and Neuroscience, King's College London, London, UK; 5 Center for Genomic Medicine, Massachusetts General Hospital, Boston, MA, USA; 6 South London and Maudsley NHS Foundation Trust, London, UK; 7 NIHR Biomedical Research Centre, London, UK; 8 Centre for Genomic and Experimental Medicine, Institute of Genetics & Molecular Medicine, Edinburgh, UK; 9 Usher Institute for Population Health Sciences and Informatics, Edinburgh, UK; 10 MRC Human Genetics Unit, Institute of Genetics and Molecular Medicine, University of Edinburgh, Edinburgh, UK

**Keywords:** Selection bias, cohort studies, mental health, follow-up studies, genome-wide association study, UK Biobank, Generation Scotland, Partners Biobank

## Abstract

**Background:**

People who opt to participate in scientific studies tend to be healthier, wealthier and more educated than the broader population. Although selection bias does not always pose a problem for analysing the relationships between exposures and diseases or other outcomes, it can lead to biased effect size estimates. Biased estimates may weaken the utility of genetic findings because the goal is often to make inferences in a new sample (such as in polygenic risk score analysis).

**Methods:**

We used data from UK Biobank, Generation Scotland and Partners Biobank and conducted phenotypic and genome-wide association analyses on two phenotypes that reflected mental health data availability: (i) whether participants were contactable by e-mail for follow-up; and (ii) whether participants responded to follow-up surveys of mental health.

**Results:**

In UK Biobank, we identified nine genetic loci associated (*P* <5 × 10^–8^) with e-mail contact and 25 loci associated with mental health survey completion. Both phenotypes were positively genetically correlated with higher educational attainment and better health and negatively genetically correlated with psychological distress and schizophrenia. One single nucleotide polymorphism association replicated along with the overall direction of effect of all association results.

**Conclusions:**

Re-contact availability and follow-up participation can act as further genetic filters for data on mental health phenotypes.

**Key Messages**

- Large cohort studies show a ‘healthy volunteer bias’ and this type of selection bias has a polygenic basis.
- Participants who take part in follow-up studies of mental health differ from participants who do not, and tend to be healthier, better educated and to have a family history of dementia and/or depression.
- Genetic factors that positively associate with follow-up survey participation are positively related to cognitive function and psychological well-being and negatively related to psychiatric disorders.

## Introduction

Selection bias in epidemiological and cohort studies occurs when characteristics of individuals that influence their likelihood of becoming or remaining as study participants are also related to exposure to risk factors or to outcomes of interest.^1^ Selection bias can be introduced at many stages of a study, including at recruitment, at follow up, during record linkage or in non-response to questionnaires or tasks and has the potential to lead to misestimates of phenotypic and genetic associations.^2^ For example, a longitudinal study of psychiatric traits identified several characteristics related to loss-to-follow-up including: age; education; ancestry; geographical location; and the presence, severity and comorbidity of anxiety and depression.^3^ There are several methods for handling selection bias if and when it needs to be taken into consideration. When all variables that influence selection and attrition are known, then bias can potentially be reduced or eliminated by conditioning on known variables or including them as predictors.^4^ In longitudinal studies, techniques such as inverse probability weighting, where observations that are similar to those that were lost to follow-up contribute proportionally more to the analysis, can be used to correct for selection bias.^5^ Given the importance of selection bias on inference, it is crucial to fully characterize it in any given study population.

Initial ascertainment and re-contact have been demonstrated to have a genetic basis. For example, individuals who had a high genetic risk of schizophrenia (calculated from polygenic risk scores) were less likely to complete follow-up questionnaires or attend additional data collection sessions,^6^ and genetic propensity for other traits have similar effects.^7^ Participation in large cohort studies is already known to have a ‘healthy volunteer’ effect,^8^ so we sought to characterize the phenotypic and genetic correlates of participation in follow-up studies focused on assessing mental health traits. To this end, we analysed re-contact and participation in three studies: the Mental Health Questionnaire (MHQ) online follow-up in UK Biobank^9^ (*N* = 371 417–373 478), the Stratifying Resilience and Depression Longitudinally (STRADL) study in Generation Scotland^10^ (*N* = 19 994) and the Partners Biobank^11^ (*N* = 15 925). We conducted phenotypic and genome-wide association analyses in UK Biobank to determine how participants who completed the MHQ differed from the rest of the sample. We also analysed factors related to whether UK Biobank participants were contactable by e-mail, as e-mail invitations were the primary method of recruitment into the MHQ follow-up. We used participation in the STRADL questionnaire follow-up in Generation Scotland and a health information survey follow-up questionnaire in the Partners Biobank as replication data sets for genetic findings.

Conducting genetic analyses of selection bias and loss-to-follow-up can complement and add to existing knowledge gained by comparing biobank cohorts with national statistics and published disease incidences and by comparing follow-up responders and non-responders on key characteristics. A participant’s decision to continue to engage in a research study is likely to be multifactorial. Genetic analyses are a pragmatic first step in indicating what the many contributing factors are, since genome-wide association summary statistics can be efficiently compared with those from hundreds of other studies.

Genetic analyses can be revealing in other ways. First, genetic and environmental factors may have different magnitudes or directions of association with follow-up participation. Thus, genetic studies of follow-up samples may differ in the degree to which they are susceptible to selection bias, compared with phenotypic studies. Second, a genetic study makes it possible to evaluate selection bias from traits that are only measured in a follow-up sample. For example, the Mental Health Questionnaire in UK Biobank includes evaluations of depression, anxiety, addiction and trauma that were not measured at baseline (so it is not possible to directly compare responders and non-responders on these traits). Comparisons between responders and non-responders can even be made for traits that are rare or not even measured in the Biobank. Genetic analyses can be correlated with external genome-wide summary statistics to elucidate the role of liability to disorders that are rare in most biobank samples, such as anorexia and schizophrenia. Finally, genetic summary statistics for follow-up response in a large sample in UK Biobank can become the basis for the analysis of selection bias in other genetic cohorts.

## Methods

### Samples

UK Biobank (UKB) is a population-based study of health in middle-aged and older individuals (*N* = 502 616). Eligible participants were aged 40 to 69 and recruited from 22 assessment centres in the UK. UK Biobank received ethical approval from the Research Ethics Committee (reference 11/NW/0382). The present study was conducted under UK Biobank application 4844.

Generation Scotland: Scottish Family Health Study (GS: SFHS) is a family-based cohort (*N* = 24 091) recruited through general practitioners in Scotland.^12^^,^^13^ Eligible participants were aged 18 years or older who were able to recruit one or more family members into the study. GS: SFHS received ethical approval from the Tayside Research Ethics Committee (reference 05/S1401/89).

Partners Biobank is a hospital-based cohort study from the Partners HealthCare hospitals with electronic medical records and genetic data supplemented with electronic health and lifestyle surveys.^11^ Recruitment started in 2010 (*N* = 78 726 in 2018) and is ongoing, participating across several clinics including Brigham and Women’s Hospital and Massachusetts General Hospital. All participants provided consent upon enrolment. The current analysis was restricted to adults aged 18 years or older and of European ancestry^14^ with high-quality genotyping data at the time of analysis.

### Re-contact and participation measures

During recruitment and baseline assessment (2006–10), UKB participants were given the option of supplying an e-mail address for receiving newsletters and invitations for online follow-up assessments. Of the 317 785 participants who supplied an e-mail address, 294 738 provided a usable one but the remaining 23 047 either provided a syntactically incorrect or non-existent e-mail address or asked that their e-mail address be withdrawn. An e-mail address was not provided by 184 831 UKB participants during baseline assessment. This variable is called ‘e-mail access’ in the UK Biobank documentation (field 20005), but we refer to this phenotype as ‘e-mail contact’. Although additional UK Biobank participants have subsequently provided an e-mail address for re-contact, here we analyse the baseline availability of e-mail contact so that it can be related to other baseline factors that were captured contemporaneously.

Starting in 2016, UKB participants who had provided e-mail contact were sent an invitation to an online Mental Health Questionnaire (MHQ) entitled ‘thoughts and feelings.’^9^ Participants who had not started the questionnaire or had only partially completed it were sent reminder e-mails after 2 weeks and again after 4 months. Participants also received information about the MHQ in a postal newsletter with instructions on how to participate. From data supplied by UK Biobank on 12 June 2018, 157 396 participants had completed the MHQ. Responses to the MHQ were submitted between July 2016 and July 2017. Mean time between baseline assessment and MHQ follow-up was 7.5 years (range 5.9–11.2 years). We refer to this phenotype as ‘MHQ data’.

In 2015, GS: SFHS participants were sent a questionnaire package by post as part of the Stratifying Resilience and Depression Longitudinally (STRADL) project, with the aim of studying psychological resilience.^10^ Participants were eligible for follow-up if they had consented to re-contact and if they had a Community Health Index (CHI) number. Of the 21 525 eligible participants, 9618 responded to the questionnaire, from which we coded a ‘STRADL data’ phenotype.

In the Partners Biobank, following enrolment, participants were invited to complete the Partners Biobank Health Information Survey, an optional online lifestyle, environment and family history survey.^14^ Of the 15 925 participants of European ancestry with genetic data at the time of analysis, 6639 responded to the questionnaire.

### Phenotype analysis

Demographic and health differences between responders and non-responders to the STRADL survey have been analysed previously and showed that, among other differences, participants who were women, non-smokers or who had low levels of psychological distress were more likely to respond. We thus first conducted a similar analysis in UK Biobank. We ran logistic regressions for e-mail contact and MHQ data using R 3.5.0.^15^ We examined associations with age at initial assessment, sex, geographical region, educational qualification, smoking, alcohol consumption, number of diagnoses in linked electronic health records and family history of dementia and depression (see Supplementary Information, available as Supplementary data at *IJE* online, for regression input coding).

### Genome-wide association, linkage disequilibrium score analysis, and replication analysis

We conducted genome-wide association studies (GWAS) on the UKB e-mail contact and MHQ data phenotypes and conducted gene-based association and gene-set analyses (see Supplementary Information, available as Supplementary data at *IJE* online). We calculated a genomic control factor (λ_GC_)^16^ for each set of GWAS results, which measures the inflation in test statistics above what would be expected by chance. Inflation in test statistics can caused both by a large number of genetic variants having an association with each trait (polygenicity) and by confounding factors, including population stratification and relatedness within the sample. We used linkage disequilibrium (LD) score regression^17^ to distinguish polygenicity from confounding. LD score regression exploits the increase in association test statistics for genetic loci that are closely linked in the region surrounding each causal genetic variant (indicating polygenicity) to distinguish from confounding, which is expected to inflate test statistics evenly across the whole genome. The intercept from an LD score regression quantifies the test statistic inflation from confounding factors, where an intercept estimate close to 1.0 indicates no confounding. We also used LD score regression to estimate the proportion of variance in these traits attributable to common genetic variants [also referred to as single nucleotide polymorphism (SNP) heritability] and calculated genetic correlations with 235 traits using LD Hub. We used false discovery rate to correct for multiple testing. To test for possible effects of mortality on loss-to-follow-up, we used the death register to identify participants whose death occurred before the MHQ assessment (*N* = 10 623). We then ran a GWAS on MHQ data with these participants removed.

In the replication data sets (Generation Scotland and Partners Biobank) we first tested for replication of independent SNPs (r^2^ = 0.1, 250 kb window) after Bonferroni correction. We calculated the expected power of replication using the Genetic Association Study power calculator.^18^ Following that, we tested for replication of direction of effect by performing a binomial test for the number of SNPs with the same direction of effect between the UK Biobank and Partners association results. We also calculated LD score genetic correlations^17^ between the UK Biobank and Generation Scotland summary statistics, to estimate genome-wide similarity in phenotypes between these studies.

## Results

### Phenotypic associations of e-mail contact and mental health follow-up (MHQ) data in UK Biobank

We conducted logistic regressions on e-mail contact (valid e-mail address provided vs no valid e-mail address provided) and MHQ participation (those that had completed the MHQ vs those that had not completed the MHQ) in UK Biobank, examining the effects of age, sex, geographical region, educational attainment, alcohol consumption, smoking status and personal and family history of disease. We retained participants with complete data for analysis (*N *=* *373 478). Odds ratios from the logistic regressions are listed in Table 1. Women in UK Biobank were less likely to have provided an e-mail address but more likely to take part in the MHQ. There was regional variation in e-mail contact and MHQ data. Individuals who attended assessment centres in Greater London and the South West of England were the most likely to have provided an e-mail address, whereas individuals from assessment centres in the North East of England and Scotland were the least likely. Individuals with greater educational attainment, those who were not current smokers, those with a fewer number of hospital diagnoses and those with a family history of dementia or severe depression were more likely to have e-mail contact and to have MHQ data.

**Table 1.** Logistic regression on e-mail contact and MHQ data in UK Biobank (*N *=* *373 478). Regression coefficients are expressed as odds ratios for increased probability of having e-mail contact and increased probability of having MHQ data

		E-mail contact			MHQ data	
	Variable	N	OR (SE)	95% CI	OR (SE)	95% CI
	Age (SD)	373 478	0.85 (0.004)	0.846–0.861	1.01 (0.004)	0.998–1.014
Sex	Female	211 768	1	−	1	−
	Male	161 710	1.11 (0.010)	1.093–1.131	0.90 (0.008)	0.883–0.914
Region	East Midlands	25 307	1	−	1	−
	Greater London	50 795	1.85 (0.032)	1.785–1.909	1.13 (0.022)	1.088–1.173
	North East	27 594	0.49 (0.008)	0.470–0.501	0.87 (0.018)	0.835–0.904
	North West	54 053	0.81 (0.013)	0.781–0.833	0.84 (0.012)	0.817–0.866
	Scotland	27 557	0.42 (0.009)	0.405–0.439	0.83 (0.017)	0.800–0.866
	South East	34 114	0.84 (0.016)	0.805–0.867	1.13 (0.020)	1.088–1.165
	South West	33 410	1.13 (0.021)	1.087–1.171	1.08 (0.020)	1.042–1.121
	Wales	15 741	0.58 (0.013)	0.558–0.611	0.83 (0.020)	0.796–0.873
	West Midlands	33 042	0.63 (0.011)	0.606–0.649	0.83 (0.016)	0.799–0.862
	Yorkshire	71 865	1.00 (0.016)	0.967–1.028	0.93 (0.014)	0.900–0.957
Qualifications	None	53 654	1	−	1	−
	GCSE	124 377	2.35 (0.028)	2.297–2.408	2.29 (0.029)	2.230–2.342
	A Levels	44 132	3.43 (0.048)	3.338–3.525	3.53 (0.057)	3.421–3.642
	Other	19 583	2.53 (0.042)	2.451–2.616	2.72 (0.052)	2.620–2.823
	College/university	131 732	4.27 (0.054)	4.163–4.375	4.43 (0.056)	4.322–4.541
Smoking	Never	210 858	1	−	1	−
	Previous	126 802	1.13 (0.009)	1.116–1.152	1.06 (0.008)	1.042–1.074
	Current	35 818	0.71 (0.009)	0.689–0.723	0.73 (0.010)	0.706–0.744
Alcohol	Units/week (SD)	373 478	1.05 (0.004)	1.038–1.053	1.03 (0.005)	1.021–1.039
Anthropometry	Body mass index (SD)	373 478	0.95 (0.004)	0.940–0.953	0.88 (0.004)	0.877–0.893
Diagnoses, yes (vs no)						
	Mental disorder	24 668	0.75 (0.011)	0.729–0.774	0.68 (0.012)	0.654–0.701
	Injury	59 706	0.90 (0.007)	0.881–0.909	0.83 (0.009)	0.815–0.851
	Other disease	278 019	0.95 (0.009)	0.929–0.963	0.91 (0.009)	0.889–0.923
Family history, yes (vs no)						
	Alzheimer's/dementia	52 238	1.18 (0.013)	1.157–1.208	1.22 (0.013)	1.198–1.250
	Severe depression	54 651	1.04 (0.011)	1.022–1.066	1.11 (0.012)	1.084–1.131

### Genome-wide association analysis of e-mail contact and MHQ data in UK Biobank

**Figure 1.**
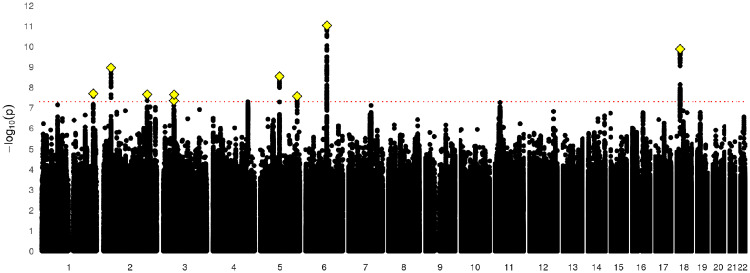
Manhattan plot of e-mail contact in UK Biobank.

**Figure 2.**
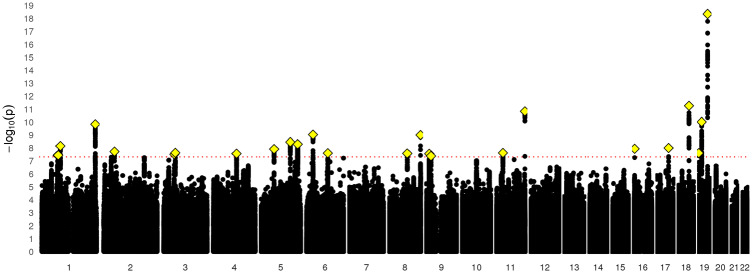
Manhattan plot of data available in MHQ follow-up.

After filtering UK Biobank individuals to a White, British, unrelated sample, the sample size was *N *=* *371 417 for the GWAS of e-mail contact and *N *=* *371 428 for the GWAS of MHQ data. After clumping, there were nine loci (*P* ≤ 5 × 10^−8^) for e-mail contact (Figure 1, Table 2; Supplementary Table S1, available as Supplementary data at *IJE* online) and 25 for MHQ participation (Figure 2, Table 3;Supplementary Table S11, available as Supplementary data at *IJE* online). The λ_GC_ was 1.29 for e-mail contact and 1.37 for MHQ data. The LD score intercept for e-mail contact and for MHQ data in UK Biobank was 1.013 [standard error (SE) 0.008] and 1.020 (SE 0.008). respectively. This yielded inflation ratios indicating that only 3.7% (SE 0.025) and 4.3% (SE 0.020) of the inflation in test statistics for e-mail contact and MHQ data was caused by confounding factors, and thus most of the inflation in test statistics was attributed to a large number of genetic loci influencing both traits (polygenicity).

**Table 2.** Top lead SNPs associated with e-mail contact in UK Biobank. Direction of effects are listed for the UK Biobank discovery sample and the Generation Scotland and Partners Biobank replication samples as either positive (+) or negative (−)

Chr	SNP	Location (bp)	A1/A2	Freq.	OR (SE)	*P*-value	Direction
1	rs632180	234, 758, 181	T/C	0.70	0.973 (0.005)	2.0 × 10^−8^	−−+
2	rs7597665	34, 420, 702	C/T	0.29	1.031 (0.005)	1.1 × 10^−9^	+++
2	rs1455343	199, 519, 691	T/G	0.38	0.974 (0.005)	2.2 × 10^−8^	−−+
3	rs73078357	48, 695, 834	C/T	0.12	1.038 (0.007)	4.5 × 10^−8^	+++
3	rs111488606	49, 864, 924	CA/C	0.44	0.973 (0.005)	2.3 × 10^−8^	−−−
5	rs6452788	87, 712, 913	A/G	0.24	1.032 (0.005)	2.9 × 10^−9^	++−
5	rs4976602	167, 843, 998	A/G	0.11	0.96 (0.007)	2.7 × 10^−8^	−−−
6	rs1487441	98, 553, 894	A/G	0.49	1.031 (0.005)	9.5 × 10^−12^	+++
18	rs1788784	21, 159, 630	G/A	0.66	1.031 (0.005)	1.3 × 10^−10^	+++

A1, effect allele; A2, non-effect allele; Chr, chromosome; Freq., frequency of effect allele; OR, odds ratio; SE, standard error.

**Table 3.** Top lead SNPs associated with MHQ data. Direction of effects are listed for the UK Biobank discovery sample and the Generation Scotland and Partners Biobank replication samples as either positive (+) or negative (−)

Chr	SNP	Location (bp)	A1/A2	Freq.	OR (SE)	*P*-value	Direction
1	rs7542974	72, 544, 704	A/G	0.25	1.032 (0.006)	3.8 × 10^−8^	+++
1	rs485929	74, 678, 285	G/A	0.39	1.028 (0.005)	3.7 × 10^−8^	+−+
1	rs532246	84, 411, 238	G/A	0.74	0.968 (0.005)	7.0 × 10^−9^	−+−
1	rs2789111	243, 346, 404	C/T	0.38	0.968 (0.005)	1.5 × 10^−10^	−−+
2	rs35028061	49, 479, 987	GT/G	0.38	1.029 (0.005)	1.9 × 10^−8^	+−−
3	rs9917656	48, 581, 513	C/T	0.30	1.03 (0.006)	3.2 × 10^−8^	++−
3	rs13082026	52, 962, 681	T/C	0.44	0.972 (0.005)	2.4 × 10^−8^	−−+
4	rs57692580	106, 214, 476	A/T	0.39	0.973 (0.005)	2.8 × 10^−8^	−++
5	rs34635	60, 513, 501	G/A	0.42	0.972 (0.005)	1.2 × 10^−8^	−−−
5	rs146681214	133, 867, 867	AC/A	0.18	1.039 (0.007)	3.6 × 10^−9^	+++
5	rs2336897	167, 050, 276	T/C	0.69	1.031 (0.005)	5.2 × 10^−9^	++−
6	rs3993747	31, 580, 507	G/A	0.35	0.969 (0.005)	9.5 × 10^−10^	−−−
6	rs59732267	98, 432, 302	CA/C	0.52	0.972 (0.005)	2.5 × 10^−8^	−−−
8	rs28716319	83, 269, 854	G/A	0.28	1.031 (0.005)	2.7 × 10^−8^	+−+
8	rs13262595	143, 316, 970	G/A	0.56	1.03 (0.005)	1.0 × 10^−9^	+++
9	rs6474966	15, 757, 537	A/G	0.46	1.028 (0.005)	2.8 × 10^−8^	+++
9	rs11793831	23, 362, 311	T/G	0.42	1.027 (0.005)	4.3 × 10^−8^	+−+
11	rs1984389	31, 740, 989	C/A	0.54	0.973 (0.005)	2.4 × 10^−8^	−−−
11	rs10791143	131, 278, 676	G/A	0.62	1.034 (0.005)	1.5 × 10^−11^	+++
16	rs4616299	7, 657, 432	G/A	0.40	0.972 (0.005)	1.2 × 10^−8^	−−−
17	rs56058331	56, 427, 128	A/G	0.42	1.029 (0.005)	1.0 × 10^−8^	+++
18	rs1261078	52, 866, 791	G/A	0.05	0.927 (0.010)	5.6 × 10^−12^	−+−
19	rs34232444	4, 965, 404	C/T	0.35	1.029 (0.005)	2.5 × 10^−8^	++−
19	rs3746187	18, 279, 816	G/A	0.40	0.968 (0.005)	9.8 × 10^−11^	−−−
19	rs429358	45, 411, 941	C/T	0.15	0.942 (0.006)	4.6 × 10^−19^	−−−

A1, effect allele; A2, non-effect allele; Chr, chromosome; Freq., frequency of effect allele; OR, odds ratio; SE, standard error.

### Loci discovery and annotation of the e-mail contact and MHQ phenotypes

The nine loci associated with e-mail contact were found to contain an over-representation of SNPs found in ncRNA intronic regions (57.5%), as well as SNPs found in intronic regions (28.4%) (Supplementary Figure S1 and Supplementary Table S1, available as Supplementary data at *IJE* online). Evidence was also found that these loci contained regulatory regions of the genome, indicated by 32.0% of the SNPs in the genomic loci having RegulomeDB (RDB) less than 2, indicating that genetic variation in these loci is likely to affect gene expression. Finally, 77.6% of the SNPs within the independent genomic loci had a minimum chromatin state of <8. This is further evidence that these loci are located in an open chromatin state and that they are located within regulatory regions. Using the GWAS catalogue, lead and tagging SNPs from these nine independent genomic loci were found to overlap with loci previously associated with body mass index and obesity (two loci), as well as with educational attainment and intelligence (three loci) (Supplementary Table S2, available as Supplementary data at *IJE* online).

The 25 loci associated with the MHQ participation phenotype notably included rs429358, a missense mutation in *APOE*. The rs429358-C allele is a marker for APOE- ε4 genotype, and the direction of the effect for this SNP indicated that participants with more copies of APOE-ε4 were less likely to participate in the MHQ [odds ratio (OR) = 0.942 ± 0.0057SE for each additional ε4 copy]. Functional annotation of the SNPs found within these regions showed that these SNPs were primarily located in introns (47.3%), and intergenic regions (17.7%) and 2.9% had no known function (Supplementary Figure S2 and Supplementary Table S8, available as Supplementary data at *IJE* online). Of these SNPs, 30.8% had an RDB score of less than 2 and 83.8% had a minimum chromatin value of less than 8, providing further evidence that these variants are located in regions of the genome that are linked to gene regulation. These 25 loci showed overlap with the loci identified in previous GWAS examining cognitive abilities and education (six loci), schizophrenia (five loci), and Alzheimer’s disease (one locus) (Supplementary Table S9, available as Supplementary data at *IJE* online).

### Gene mapping of the e-mail access and MHQ phenotype

We used three strategies for mapping the SNPs in the associated loci to genes. First, positional mapping aligned the SNPs from the independent genomic loci associated with e-mail contact to 20 genes by using location, whereas eQTL mapping matched cis-eQTL SNPs to 40 genes whose level of expression they have been shown to influence. Finally, chromatin interaction mapping annotated SNPs to a total of 41 genes, using three-dimensional DNA-DNA interactions between the SNPs’ genomic regions, and close or distant genes (Supplementary Tables S4 and S5, Supplementary Figure 5a–f, available as Supplementary data at *IJE* online). Collectively these mapping strategies identified 70 unique genes, of which 21 were implicated by two mapping strategies and 10 were implicated by all three. A total of five genes, *TNNI3K*, *LRRIQ3*, *NEGR1*, *FPGT* and *FPGT-TNNI3K*, were implicated using all three methods and showed evidence of a chromatin interaction between two independent genomic risk loci (Supplementary Table S4, available as Supplementary data at *IJE* online). Gene-based statistics derived in MAGMA indicated a role for 72 genes (Supplementary Table S5, available as Supplementary data at *IJE* online), four of which overlapped with genes implicated by all three mapping strategies (Supplementary Figure S3, available as Supplementary data at *IJE* online).

For the MHQ data phenotype, positional mapping implicated 42 genes, with eQTL mapping indicating a role for 86 genes. Chromatin interaction mapping annotated a total of 124 genes (Supplementary Tables S14 and S15, Supplementary Figure S6a–m, available as Supplementary data at *IJE* online). Across these three mapping strategies, 181 unique genes were identified, with 46 of these being implicated by two mapping strategies and 25 being implicated by all three. MAGMA was also used and indicated a role for 81 genes (Supplementary Figure S4 and Supplementary Table S15, available as Supplementary data at *IJE* online). Fifteen of these 81 genes overlapped with those identified using the three mapping strategies.

### Gene-set and gene property analysis

The presynaptic membrane gene-set was enriched for the e-mail contact phenotype (*P* = 5.19 × 10^−7^) (Supplementary Table S6, available as Supplementary data at *IJE* online). Gene property analysis showed a relationship between expression in the Epstein-Barr virus (EBV)-transformed lymphocyte cells (*P* = 9.24 × 10^−4^) and for gene expression in the early mid-prenatal time of life (*P* = 0.004) (Supplementary Tables S9 and S10, available as Supplementary data at *IJE* online).

For the MHQ data phenotype none of the gene sets were enriched (Supplementary Table S16, available as Supplementary data at *IJE* online). However, gene property analysis indicated a relationship between gene expression in the brain and the MHQ phenotype (*P* = 2.64 × 10^−4^) (Supplementary Table S17, available as Supplementary data at *IJE* online). When examining the specific tissue gene groupings, this relationship was driven by expression change in the cerebellar hemisphere (*P* = 8.52 × 10^−6^) and the cerebellum (*P* = 1.27 × 10^−5^) (Supplementary Table S18, available as Supplementary data at *IJE* online). A relationship between gene expression in the early prenatal lifespan (*P* = 0.002) and the early mid-prenatal lifespan was also found (*P* = 5.33 × 10^−4^) (Supplementary Table S19, available as Supplementary data at *IJE* online).

### LD score regression analysis

We used LD score regression to estimate SNP heritability from the GWAS results. Heritability on the liability scale for e-mail contact was 0.073 (0.004 SE) and for MHQ data was 0.099 (0.004 SE). The genetic correlation between e-mail contact and MHQ data was 0.822 (0.020 SE).

**Figure 3.**
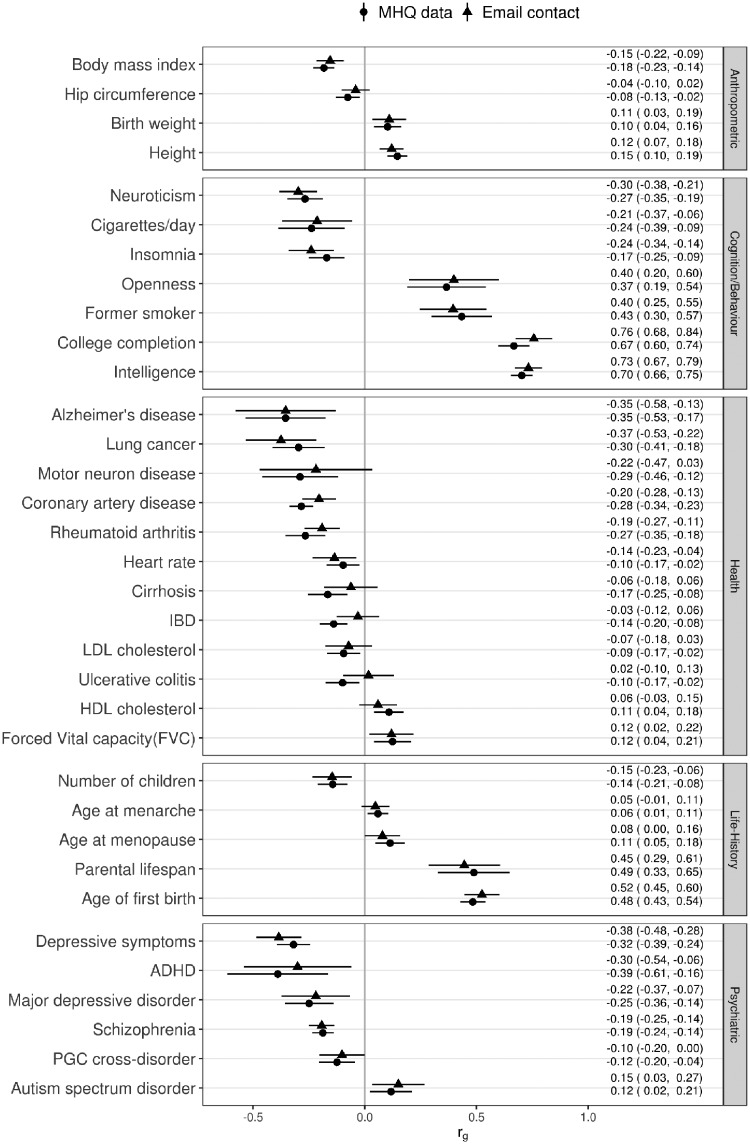
LD score genetic correlations (rg) with e-mail contact (triangle) and MHQ data (circle), with 95% confidence intervals.

We used LD Hub^19^ to estimate genetic correlations with a large number of other traits. Both e-mail contact and having MHQ data were genetically correlated with a broad spectrum of traits. Results for an illustrative set of traits are plotted in Figure 3 and the results for all traits are listed in Supplementary Table S21, available as Supplementary data at *IJE* online. For most anthropometric, behavioural, cognitive, psychiatric, health-related and life history traits the direction of the genetic correlations with e-mail contact and MHQ participation was the same. In general, genetic factors associated with providing an e-mail address for re-contact to UK Biobank and taking part in the MHQ were also associated with better health, higher intelligence, lower burden of psychiatric disorders and a slower life-history (e.g. later age at menarche, age at first birth and age at menopause). Both e-mail contact and MHQ participation were not genetically correlated with any traits categorized as bone, kidney, uric acid and metals (transferrin/ferritin). Additionally, e-mail contact was not genetically correlated with glycaemic traits and MHQ data availability was not genetically correlated with hormone or metabolite phenotypes.

### Effect of mortality on MHQ genetic associations

To test for the role of mortality on our findings, we re-ran the genome-wide association analysis of MHQ data availability after removing participants whose dates of death occurred before the MHQ assessment. The overall inflation in association test statistics including and excluding deceased participants was identical (mean χ^2^ = 1.438) and the genetic correlation between the two sets of summary statistics was 0.9996 (SE = 0.0002). We compared the top independent associated SNPs in the GWAS in the larger sample with those that excluded deaths (Supplementary Table S24 and Figure S7, available as Supplementary data at *IJE* online). Although there were three SNPs that no longer passed the criterion for genome-wide significance, there was no appreciable change in the effect sizes estimates for any of the SNPs

### Replication in generation Scotland and partners Biobank

We examined whether any of the associations results for the e-mail and MHQ data phenotypes replicated in an independent sample, using whether members of Generation Scotland participated in the STRADL follow-up of mental health. At an alpha criterion of 0.05/34 and an average genotype relative risk of 1.015, there was 4% power to replicate in Generation Scotland and 2% power in Partners Biobank, and replicating the UK Biobank findings requires approximately 200 000 cases and controls to achieve 90% power.^18^ None of the independent SNPs in the UKB GWASs replicated in Generation Scotland after Bonferroni correction (34 tests) (Supplementary Tables S22 and S23, available as Supplementary data at *IJE* online). We observed replication evidence for one independent SNP (rs9917656, 6.2 × 10^–4^) in Partners Biobank after Bonferroni correction (Supplementary Tables S22 and S23, available as Supplementary data at *IJE* online). Between UK Biobank and Partners Biobank, more of the SNPs for survey participation had the same direction of effect than expected (20/25, exact binomial test *P*-value = 0.002). Furthermore, the STRADL data phenotype was moderately genetically correlated with both UKB e-mail contact (*r_g_* = 0.430, SE = 0.112, *P *=* *0.0001) and UKB MHQ data (*r_g_* = 0.619, SE = 0.130, *P *=* *1.98 × 10^–6^) and had an SNP heritability on the liability scale of 0.112 (SE 0.0408).

## Discussion

Using data from UK Biobank, we found that individuals who provided an e-mail address for re-contact and who participated in follow-up surveys of mental health differed from those who did not with regards to demographic, psychological, health, lifestyle and genetic factors. The UK Biobank sample differs from the UK population,^20^ and our results show that ascertainment processes also exert an effect on follow up assessments. Most of the phenotypic and genetic associations were in the same direction. These results were not due to population stratification, as only 4% of the inflation in GWAS statistics could be attributed to factors other than polygenic heritability. Having greater educational attainment, being a non-smoker or a former smoker, having fewer hospital diagnoses of illness or injury and having a family history of dementia or a family history of serious depression all predicted greater likelihood of providing e-mail contact information. Furthermore, those variables were also associated with providing responses to the online Mental Health Questionnaire (MHQ). Importantly for understanding the composition of the MHQ subset, having an inpatient diagnosis of a mental disorder was associated with lower participation rates in the MHQ [OR = 0.68, 95% confidence interval (CI) = 0.65-0.70], and this was a larger effect size than other hospital diagnoses, specifically injury (OR = 0.83) and non-psychiatric disorders (OR = 0.91). A few effects went in the opposite direction between the e-mail contact and MHQ data variables, with men and younger individuals more likely to provide an e-mail address to UK Biobank, whereas women were more likely to provide MHQ data.

E-mail contact and MHQ data availability had SNP heritabilities of 7.3% and 9.9%, respectively. We identified nine independent SNPs associated with e-mail contact and 25 for MHQ data, more than for many GWAS studies of disease traits in the same sample. Loci for both phenotypes were mostly located within regulatory regions. Of particular interest was the association of MHQ data availability with the apolipoprotein E (APOE) ε4 genotype that is a major risk factor for Alzheimer's disease.^21^ One SNP associated with MHQ data replicated in the Partners Biobank sample. The SNP, rs9917656, is in an intron in the 6-phosphofructo-2-kinase/fructose-2, 6-biphosphatase 4 (PFKFB4), a signally enzyme involved in switching between different forms of carbohydrate metabolism.^22^ However, several other genes are implicated in this locus by positional mapping (genomic locus 6 in Supplementary Table S13, available as Supplementary data at *IJE* online). Given the effect sizes found in the discovery sample, both Generation Scotland and Partners were underpowered for replicating association results. However, the consistent directions of effect in the Partners cohort and the strong genetic correlation between STRADL participation and the e-mail contact and MHQ data phenotypes, suggest that similar genetic factors are driving participation in follow-up studies.

E-mail contact and MHQ data shared similar genetic correlations with other traits. There were strong genetic correlations between e-mail contact and indicators of cognitive ability (college completion, *r_g_* = 0.76; intelligence, *r_g_* = 0.73). Contact and data availability were also genetically associated with a lower burden of genetic risk for mental illness and lower BMI. These results were in the same direction as the phenotypic analysis. The negative genetic correlation with schizophrenia matches results from follow-up participation in the ALSPAC cohort using polygenic risk scores,^6^ and suggests that this association is not specific to schizophrenia.

The similarity in the results for phenotypic and genetic factors associated with e-mail contact and MHQ data shows that the availability of an individual to be contacted by e-mail, and their choice to participate, both act as a filter for selection into the subsample of UK Biobank with Mental Health Questionnaire data. Notably, self-reports of a family history of dementia and a family history of severe depression were more common in e-mail providers and MHQ completers, but individual genetic associations with both these disorders showed negative correlations. Individuals who reported dementia or severe depression in their family were therefore more likely to be MHQ participants, even though having a personal genetic predisposition to these disorders may also decrease their likelihood of participating. Knowledge of family history may be a strong motivational factor for participating in follow-up surveys of mental health.

Our sample was large enough that we were able to identify specific genetic loci that were related to participation in follow-up studies of mental health. We were also able to analyse the genetics of one particular factor (the availability of e-mail contact for receiving invitations) that is heavily involved in the specific process of follow-up participation. However, a limitation of our analysis is that information on e-mail contact was available for participants at baseline only, and thus did not distinguish the entire subset of participants who would have received an e-mail invitation. Another limitation is that information from electronic health records only covered hospital admissions and thus would underestimate associations with milder health conditions. Our study also does not address factors that would differentially influence participation of individuals of non-European ancestry.

**Figure 4.**
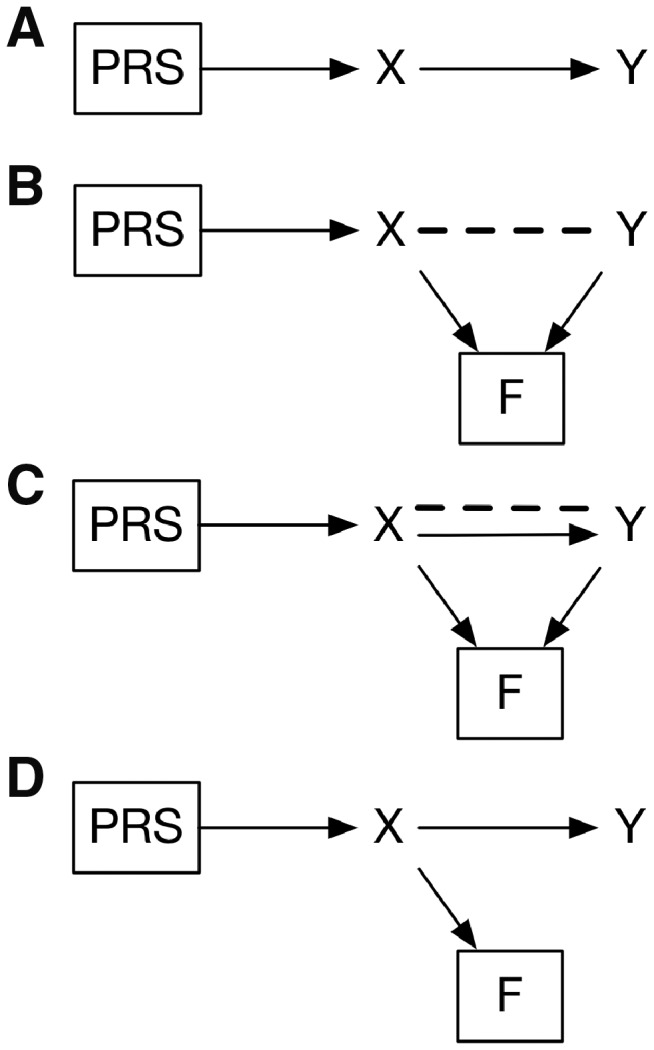
Possible effects of selection bias on polygenic risk score analyses in follow-up studies. (A) Causal model to be tested where PRS causes phenotype Y via phenotype X. (B) Worst-case scenario where PRS influences X but not Y and both phenotypes cause follow-up participation. Analysing only follow-up participants is the same as conditioning on F, which induces a correlation between PRS and Y. (C) More likely scenario, where both X and Y cause follow-up participation. Conditioning on F attenuates estimates of the relationship between PRS and Y. (D) Ideal scenario where X causes follow-up participation, but Y does not. Conditioning on F has no impact on the dependence of Y on PRS. PRS, polygenic risk score; X and Y, phenotypes of interest; F, selection into follow-up; directional solid line, true causal association; dashed line, induced or attenuated statistical dependence.

Individuals in large epidemiological cohorts who participate in follow-up surveys differ in their patterns of phenotypic and genetic association with traits of interest from those who do not. Because most factors had a consistent relationship with the two-step selection process (contactability by e-mail and opting to participate in follow-up), it is likely that these same factors may also differentiate people who choose to become part of the cohort in the first place from other people in the larger population. These factors are very likely to bias the selection of individuals for inclusion in population-based studies towards those with positive family histories but lower personal genetic risk of mental health conditions such as depression and dementia. Analysing variables within a follow-up study may have the effect inducing statistical dependence or attenuating estimates of the relationships among variables.^2^Figure 4a illustrates a hypothesized causal model where a polygenic risk score (PRS) influences a phenotype or outcome *Y* via an intermediate phenotype *X*. This model could be tested by d-separation^23^: if the model is true, then regressing *Y* on *X* will result in conditional independence of PRS and *Y*. Figure 4b illustrates a scenario analysing the effect of the PRS where participation in follow-up is a collider for the two phenotypes when they do not have a causal relationship with each other. Analysing data only within the follow-up sample creates non-independence between the *X* and *Y* traits and thus between PRS and *Y*. Even when one trait causes the other, conditioning on follow-up participation can bias the estimate of PRS on the downstream trait (Figure 4c). A scenario where only one of the traits causes follow-up would not result in biased estimates of the effects of PRS (Figure 4d).

Going forward, studies should evaluate (e.g. using simulations^2^) the particular effects that selection and attrition might have on effect estimates and, where available, check results from follow-up assessments against those from baseline data, even in the cases where the follow-up data provide better or more comprehensive measures of phenotypes of interest. Because continued participation in large cohorts studies recapitulates the ‘healthy volunteer’ effect, comparing responders and non-responders in follow-up surveys may be a useful way to analyse how selection bias may influence the generalizability and accuracy of findings.

## Data availability

GWAS summary statistics for the UK Biobank e-mail contact and Mental Health Questionnaire data are available from [https://doi.org/10.7488/ds/2554]. Underlying study data are available to bona fide researchers from UK Biobank [https://www.ukbiobank.ac.uk/], Generation Scotland [http://www.generationscotland.org/] and Partners HealthCare Biobank [https://biobank.partners.org].

## Funding

M.J.A. and A.M.Mc. are supported by MRC Mental Health Data Pathfinder award (reference MC_PC_17209) and the Wellcome Trust Strategic Award ‘STratifying Resilience and Depression Longitudinally’ (STRADL) (reference 104036/Z/14/Z). Analysis conducted under UK Biobank application 4844. W.D.H. is supported by a grant from Age UK (Disconnected Mind Project). I.J.D. is supported by the Centre for Cognitive Ageing and Cognitive Epidemiology, which is funded by the Medical Research Council and the Biotechnology and Biological Sciences Research Council (reference MR/K026992/1). D.M.H. is supported by a Sir Henry Wellcome Postdoctoral Fellowship (reference 213674/Z/18/Z) and a 2018 NARSAD Young Investigator Grant from the Brain & Behavior Research Foundation (ref: 27404). K.A.S.D. and M.H. are supported by NIHR Biomedical Research Centre at South London and Maudsley NHS Foundation Trust and King's College London. Generation Scotland received core support from the Chief Scientist Office of the Scottish Government Health Directorates (CZD/16/6) and the Scottish Funding Council (HR03006). Genotyping of the GS: SFHS samples was carried out by the Genetics Core Laboratory at the Wellcome Trust Edinburgh Clinical Research Facility, University of Edinburgh, Scotland and was funded by the Medical Research Council UK and the Wellcome Trust [Wellcome Trust Strategic Award ‘Stratifying Resilience and Depression Longitudinally’ (STRADL), reference 104036/Z/14/Z].

## Supplementary Material

dyz134_Supplementary_Data
