# Supplementary material for: Factors associated with sharing e-mail information and mental health survey participation in large population cohorts
Source: Int J Epidemiol. 2019 Jul 1;49(2):410–21. doi: 10.1093/ije/dyz134 (PMC7266553; doi:10.1093/ije/dyz134)
Supplement: dyz134_Supplementary_Data [file dyz134_supplementary_data.zip › dyz134-suppl_data/ije-2018-12-1551-File009.docx]

Factors associated with sharing email information and mental health survey participation in large population cohorts

Supplementary Information

**Logistic regression analysis input coding**

We centered and standardized age. We determined geographic region by grouping the assessment centres together into regions of England (South East, South West, East Midlands, West Midlands, North West, North East, and Yorkshire), Greater London, Scotland, and Wales. Education, smoking, drinking, and family history were assessed by means of a touchscreen interview during the initial assessment. We categorized educational qualifications as None, Professional, Higher (college or university), Secondary (A levels, O levels, GCSEs, CSEs), and Vocational (NVQ, HND, HNC). Smoking history had the responses 'Prefer not to answer', 'Never', 'Previous', and 'Current'. For alcohol drinking, participants reported their average weekly and monthly consumption for different drink types from which we derived a measure of average alcohol consumption in units per week (Clarke et al., 2017) and standardized this variable for input into the model. For linked hospital records, we first removed diagnoses related to pregnancy (ICD-10 chapter O), congenital conditions (chapter Q), and health care provision (chapters U and Z). For the remaining diagnoses, we categorized them into mental health conditions and addictions (chapter F), injuries (chapter S, T, V, and Y), and all other diseases. Participants were assigned a value of 1 for each category if they had any diagnostic codes in that category. Participants with linked hospital records who did not have any incidences of a diagnostic category were assigned a count of 0.

**Genotyping, genomic QC, and GWAS**

UK Biobank contains genotype data imputed to ~92 million variants (Bycroft et al., 2018). We performed QC procedures on SNPs with filters for MAF > 0.001 and INFO > 0.1. We removed participants who had failed genotype platform QC, who did not cluster genetically as White British, or who overlapped with Psychiatric Genomics Consortium MDD and Generation Scotland participants; and we conducted additional filtering on related individuals (Howard et al., 2018). This resulted in 16 367 095 variants for 371 428 individuals for genetic analysis (Supplementary Figure S8). We conducted genome-wide association analyses using BGENIE v1.3 (Bycroft et al., 2018) that coded the outcome variables as 0/1 in a linear regression. We covaried for age, sex, assessment centre, genotyping platform, and 20 UKB-provided principal components. We approximated odds ratios for the SNP effects using the transformation to the log-odds scale, $\text{log(OR)}=\beta/{(k (1 - k))}$, where *k* is the fraction of participants who were coded as 1 in the outcomes variable (email contact *k* = 0.6, MHQ data *k* = 0.33).

For Generation Scotland, 8 642 105 imputed variants were available for 19 994 participants (Hall et al., 2018). Variants with MAF < 0.005 and INFO < 0.8 were excluded. We performed association tests on the STRADL data phenotype using the mixed linear model with candidate marker excluded (MLMe) approach in GCTA v1.91.1 (Yang, Zaitlen, Goddard, Visscher, & Price, 2014). We constructed two GRMs using a leave-one-chromosome-out (LOCO) approach: one GRM that included all relationship coefficients and a second GRM that set relatedness to 0 when the relationship coefficients < 0.025 (Zaitlen et al., 2013). We fitted age and sex as covariates. To see if the results from the UKB phenotypes replicated, we looked up each independent significant SNP (or an LD proxy) in the GWAS of the STRADL data phenotype and assessed whether they were significant after Bonferroni correction. We also calculated the LD score genetic correlation of the STRADL data phenotype with the UKB email and MHQ data phenotypes.

For Partners Biobank, DNA from participants was genotyped using ~1.6 million SNPs on the Illumina Multi-Ethnic GWAS/Exome SNP Array and imputed using Minimac3 using the HRC (Version r1.1 2016) reference panel (Dashti, Redline, & Saxena, 2018). Replication was sought for the 35 identified signals (or an LD proxy). Individual SNPs association analyses were conducted using logistic regression analyses and an additive genetic model in PLINK adjusted for age, sex, genotyping array, and principal components of ancestry. Associations were considered significant after Bonferroni correction.

**Loci discovery and functional annotation**

Genomic risk loci were derived using clumping, carried out in FUnctional Mapping and Annotation of genetic associations (FUMA) (Watanabe, Taskesen, van Bochoven, & Posthuma, 2017). First, FUMA was used to identify independent significant SNPs using the *SNP2GENE* function. SNPs with a P-value of ≤ 5 ×10^−8^ and independent of other genome wide significant SNPs at r^2^ < 0.6 were identified from the summary GWAS statistics of the UKB email contact and MHQ data phenotypes. Second, using these independent significant SNPs, candidate SNPs were identified as all SNPs that had a MAF > 0.001 and were in LD of ≥ r^2^ 0.6 with at least one of the independent significant SNPs. These candidate SNPs included those from the UK10K/1000G and the haplotype reference consortia panel (UK Biobank release 1) and may not have been included in the UKB GWASs. Third, lead SNPs were identified using the independent significant SNPs. Lead SNPs were defined as SNPs that were independent from each other at r^2^ 0.1. Finally, genomic risk loci that were 250kb or closer were merged to form a single locus.

The lead SNPs identified above, and those in LD with the lead SNPs, were then mapped to genes using ANNOVAR and the Ensemble genes build 85. Intergenic SNPs were mapped to the two closest up- and downstream genes which can result in them being assigned to multiple genes. eQTL mapping was performed using each independent significant SNP and those in LD with it. Those SNP-gene pairs that were not significant (FDR ≤ 0.05) were omitted from the analysis.

**Gene-mapping**

Genetic variation in each of the independent genomic loci was mapped to genes using three complementary strategies. First, positional mapping was used to map SNPs to genes based on physical distance. SNPs within a 10kb window from the known protein genes found in the human reference assembly (hg19). Second, expression quantitative trait loci (eQTL) mapping was carried out by mapping SNPs to genes if allelic variation at the SNP was associated with expression levels of the gene. For eQTL mapping information on 45 tissue types from three data bases (GTEx, Blood eQTL browser, BIOS QTL browser) based on cis-QTLs where a SNPs are mapped to genes up to 1Mb away. A false discovery rate (FDR) of 0.05 was used as a cut off to define significant eQTL associations.

Finally, chromatin interaction mapping was carried out to map SNPs to genes when there is a three-dimensional DNA-DNA interaction between the independent genomic risk loci with a gene region. Chromatin interactions can involve long-range interactions between SNPs with genes as such no genomic distance boundary is applied. Hi-C data of 14 tissue types was used for chromatin interaction mapping. Chromatin interactions can also span multiple genes, and SNPs can be located in a region that interacts with other regions also containing multiple genes. In order to both reduce the number of genes mapped, and to increase the probability that those genes mapped are biologically linked to genetic variation at the independent genomic loci, only genes where one region involved with the interaction overlapped with a predicted enhancer region in any of the 111 tissue/cell types found in the Roadmap Epigenomics Project (Bernstein et al., 2010), and the other region was located in a gene promoter region (250bp upstream and 500bp downstream of the transcription start site and also predicted to be a promoter region by the Roadmap Epigenomics Project) were included here. An FDR of 1×10^−5^ was used to define a significant interaction.

**Gene-based GWAS**

Gene-based analyses have been shown to increase the power to detect association due to the multiple testing burden being reduced, in addition to the effect of multiple SNPs being combined. Gene-based GWAS was conducted using MAGMA (de Leeuw, Mooij, Heskes, & Posthuma, 2015), also implemented in FUMA (Watanabe et al., 2017). Regardless of P-value, all SNPs located within protein coding genes were used to derive a P-value describing the association between genetic variation across the gene with either email or questionnaire. The NCBI build 37 was used to determine the location and boundaries of 18 877 autosomal genes and linkage disequilibrium within and between genes was gauged using the UK Biobank 1 reference panel. A Bonferroni correction was applied to control for the number of genes tested.

**Gene-set analysis**

A competitive gene-set analysis was conducted in MAGMA to identify the biological systems vulnerable to perturbation by common genetic variation. Competitive testing examines if genes within the gene set are more strongly associated with the trait of interest than genes from outside the gene set, and differs from self-contained testing by controlling for type 1 error rate as well as being able examine the biological relevance of the gene-set under investigation.

A total of 10 894 gene-sets (sourced from Gene Ontology, Reactome, and, MSigDB) were examined for enrichment. To control for the 10,894 gene sets examined, a Bonferroni correction was applied.

Bernstein, B. E., Stamatoyannopoulos, J. A., Costello, J. F., Ren, B., Milosavljevic, A., Meissner, A., . . . Thomson, J. A. (2010). The NIH Roadmap Epigenomics Mapping Consortium. *Nature Biotechnology, 28*, 1045. doi:10.1038/nbt1010-1045

Bycroft, C., Freeman, C., Petkova, D., Band, G., Elliott, L. T., Sharp, K., . . . Marchini, J. (2018). The UK Biobank resource with deep phenotyping and genomic data. *Nature, 562*(7726), 203-209. doi:10.1038/s41586-018-0579-z

Clarke, T.-K., Adams, M. J., Davies, G., Howard, D. M., Hall, L. S., Padmanabhan, S., . . . McIntosh, A. M. (2017). Genome-wide association study of alcohol consumption and genetic overlap with other health-related traits in UK Biobank (N=112 117). *Molecular psychiatry, 22*, 1376. doi:10.1038/mp.2017.153

Dashti, H. S., Redline, S., & Saxena, R. (2018). Polygenic risk score identifies associations between sleep duration and diseases determined from an electronic medical record biobank. *Sleep*, zsy247-zsy247. doi:10.1093/sleep/zsy247

de Leeuw, C. A., Mooij, J. M., Heskes, T., & Posthuma, D. (2015). MAGMA: Generalized Gene-Set Analysis of GWAS Data. *PLOS Computational Biology, 11*(4), e1004219. doi:10.1371/journal.pcbi.1004219

Hall, L. S., Adams, M. J., Arnau-Soler, A., Clarke, T.-K., Howard, D. M., Zeng, Y., . . . Major Depressive Disorder Working Group of the Psychiatric Genomics, C. (2018). Genome-wide meta-analyses of stratified depression in Generation Scotland and UK Biobank. *Translational Psychiatry, 8*(1), 9. doi:10.1038/s41398-017-0034-1

Howard, D. M., Adams, M. J., Shirali, M., Clarke, T.-K., Marioni, R. E., Davies, G., . . . McIntosh, A. M. (2018). Genome-wide association study of depression phenotypes in UK Biobank identifies variants in excitatory synaptic pathways. *Nature communications, 9*(1), 1470. doi:10.1038/s41467-018-03819-3

Watanabe, K., Taskesen, E., van Bochoven, A., & Posthuma, D. (2017). Functional mapping and annotation of genetic associations with FUMA. *Nature communications, 8*(1), 1826. doi:10.1038/s41467-017-01261-5

Yang, J., Zaitlen, N. A., Goddard, M. E., Visscher, P. M., & Price, A. L. (2014). Advantages and pitfalls in the application of mixed-model association methods. *Nat Genet, 46*(2), 100-106. doi:10.1038/ng.2876

Zaitlen, N., Kraft, P., Patterson, N., Pasaniuc, B., Bhatia, G., Pollack, S., & Price, A. L. (2013). Using Extended Genealogy to Estimate Components of Heritability for 23 Quantitative and Dichotomous Traits. *PLOS Genetics, 9*(5), e1003520. doi:10.1371/journal.pgen.1003520
