# Supplementary material for: Factors associated with sharing e-mail information and mental health survey participation in large population cohorts
Source: Int J Epidemiol. 2019 Jul 1;49(2):410–21. doi: 10.1093/ije/dyz134 (PMC7266553; doi:10.1093/ije/dyz134)

Factors associated with sharing email information and mental health survey participation in large population cohorts

Supplementary Figures

Figure S1. Functional categories, RDB scores, and minimum chromatin states for independent risk loci associated with UKB email contact.

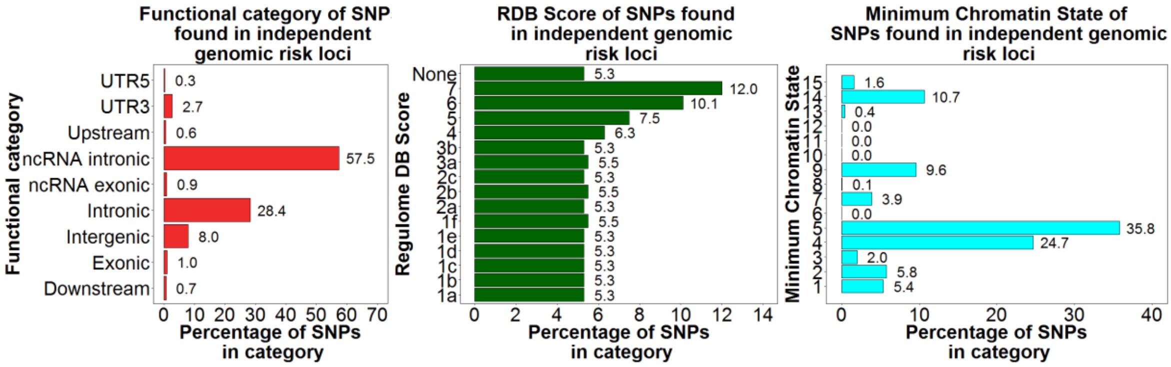

Figure S2. Functional categories, RDB scores, and minimum chromatin states for independent risk loci associated with UKB MHQ participation.

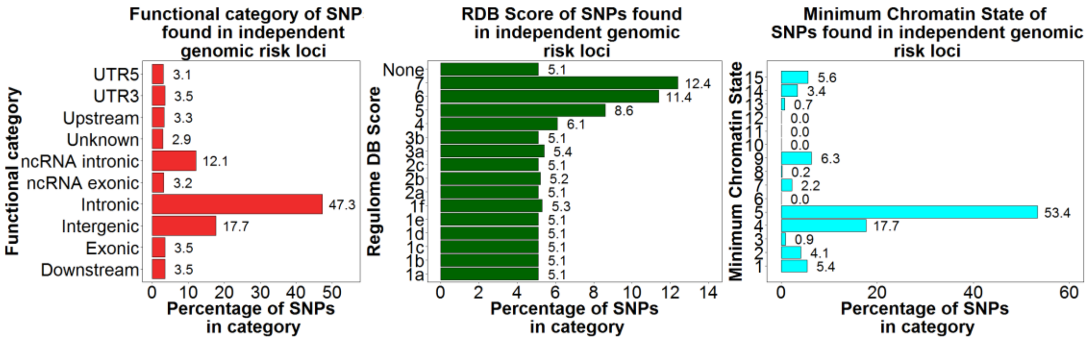

Figure S3. Number of genes implicated by different mapping strategies for UKB email contact.

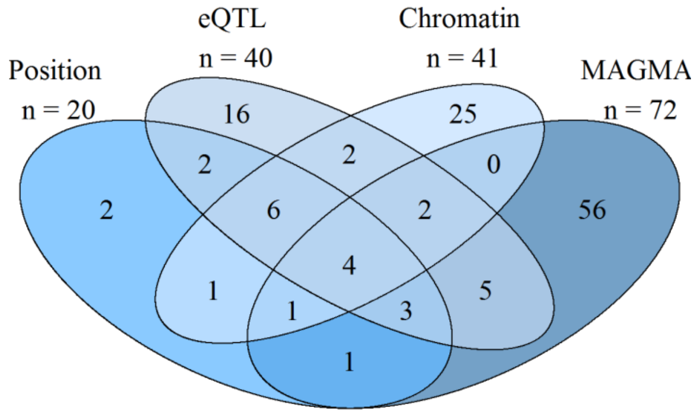

Figure S4. Number of genes implicated by different mapping strategies for UKB MHQ data.

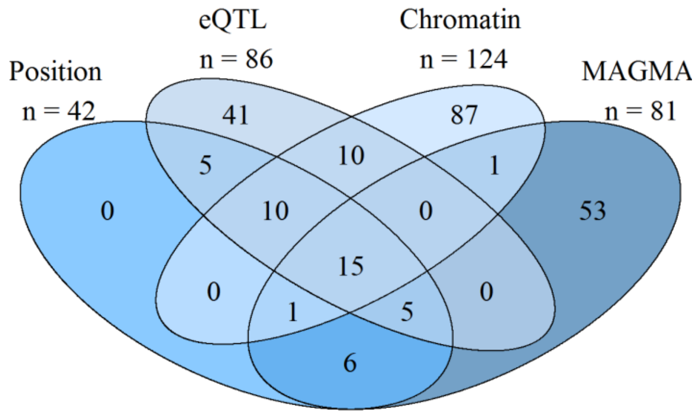

Supplementary Figures S5 and S6.

Circos plots by chromosome illustrating genome-wide significant loci associated with the Email contact and the MHQ data phenotype are shown. For each phenotype the most outer layer shows the Manhattan plot and only SNPs where P <0.05 are shown. Each of the SNPs in the genomic risk loci are colour coded indicating the maximum r^2 with one of the independent significant SNPs in the locus with red indicating the highest r^2 and blue the lowest r^2(red r^2>0.8, orange r^2>0.6, green r^2>0.4, and blue r^2>0.2). SNPs shown in grey are not in LD with any of the genome wide significant SNPs. The rsID of the most significant lead SNP in each loci is shown. The second layer is the chromosomal ring with the independent genomic risk loci highlighted in blue. Next, the genes mapped by chromatin interactions or eQTLs are displayed. Genes mapped using chromatin interactions the gene is displayed in

orange, with genes mapped by eQTL shown in green. Genes that are displayed in red are those mapped using both chromatin interactions and eQTLs. Chromatin interaction links (coloured orange for chromatin interactions and green for eQTLs are displayed.

Figure S5a. Circos plot for email contact chromosome 1

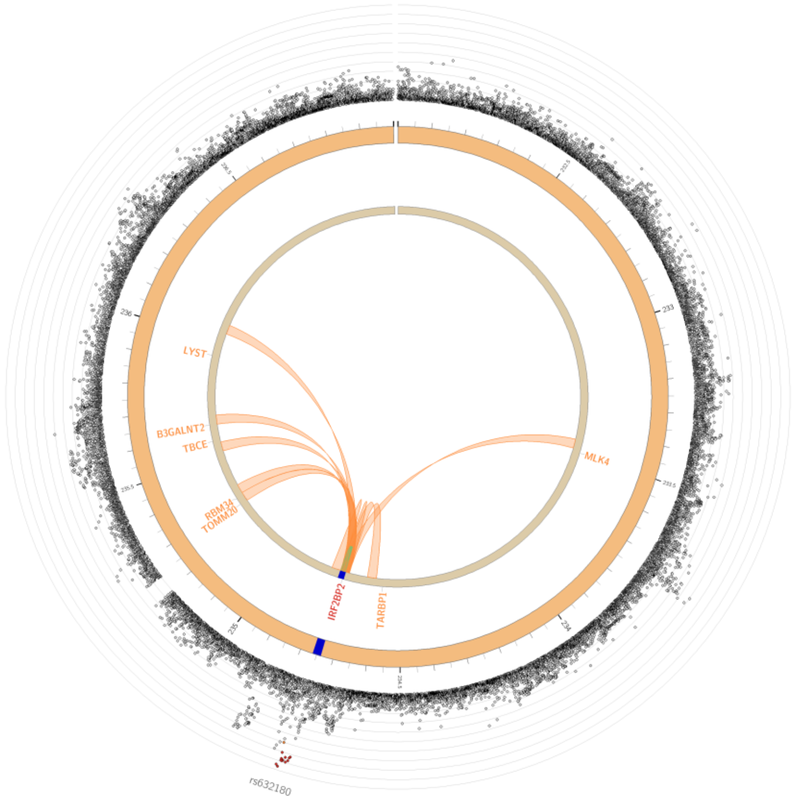

Figure S5b. Circos plot for email contact chromosome 2

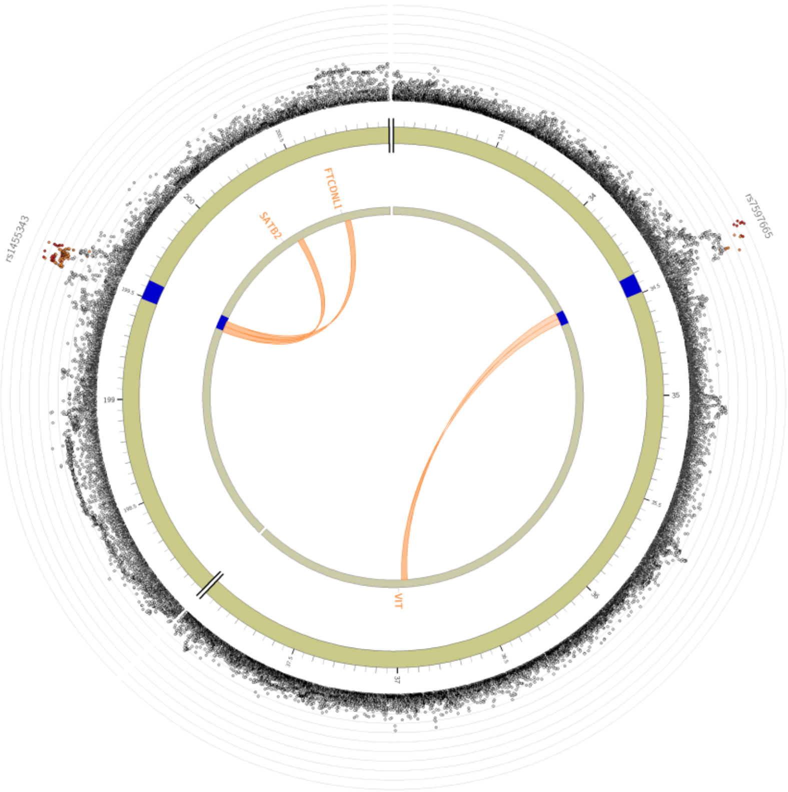

Figure S5c. Circos plot for email contact chromosome 3

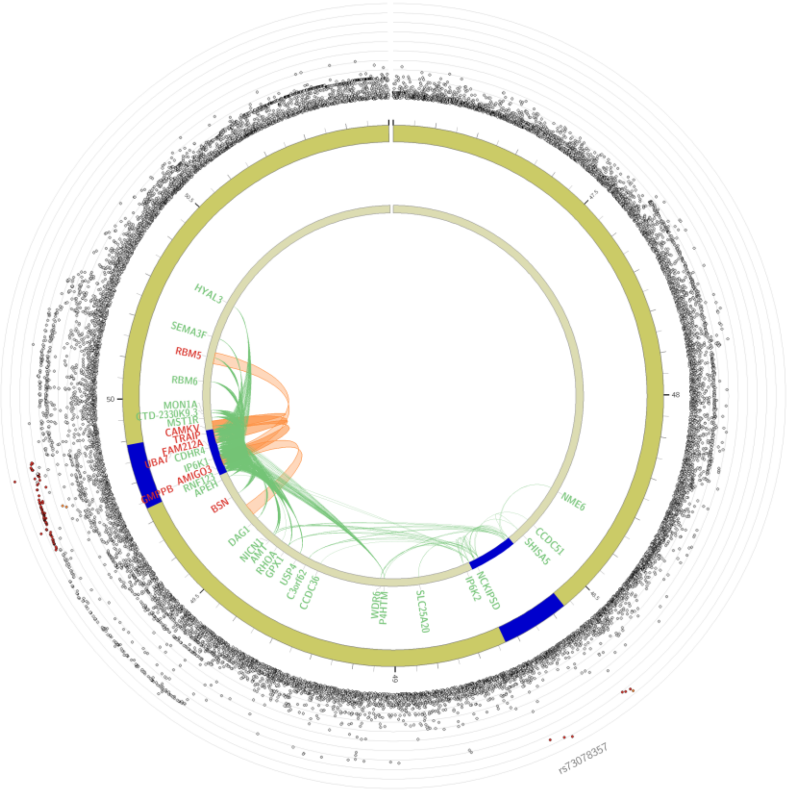

Figure S5d. Circos plot for email contact chromosome 5

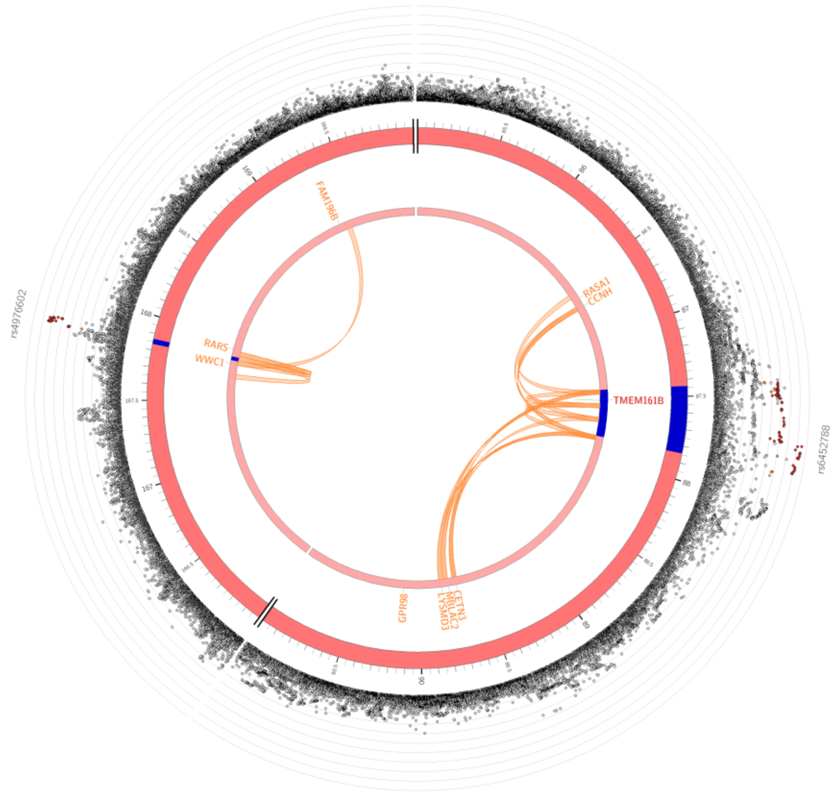

Figure S5e. Circos plot for email contact chromosome 6

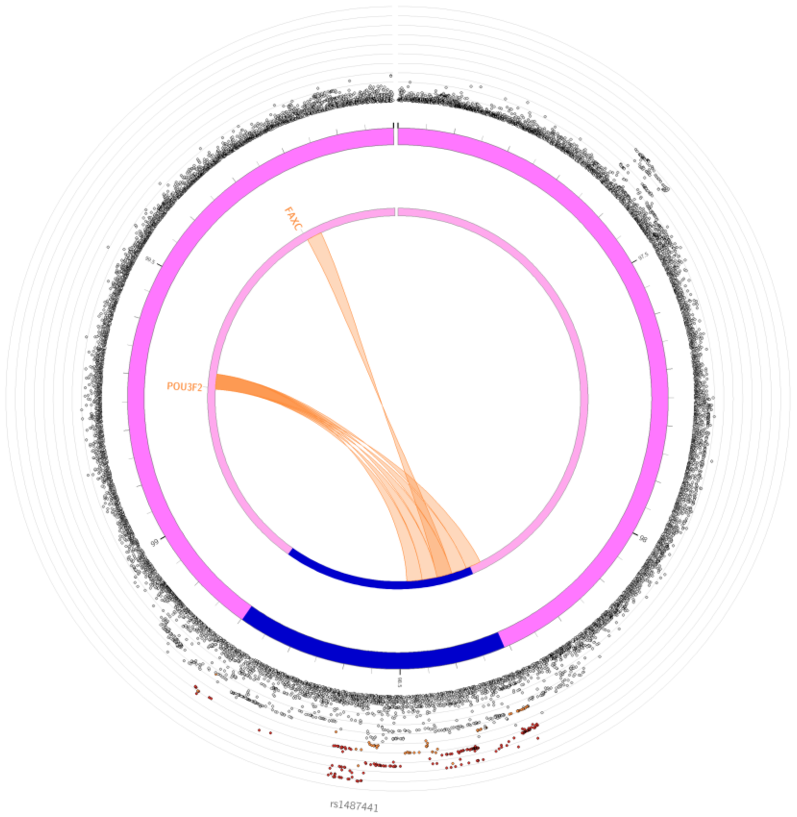

Figure S5f. Circos plot for email contact chromosome 18

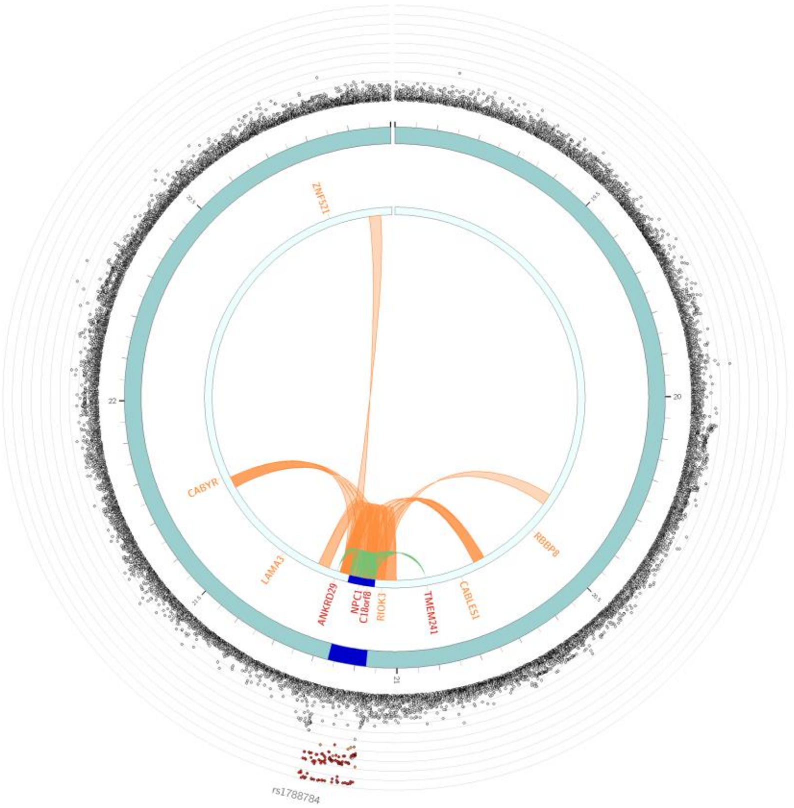

Figure S6a. Circos plot for MHQ data chromosome 1

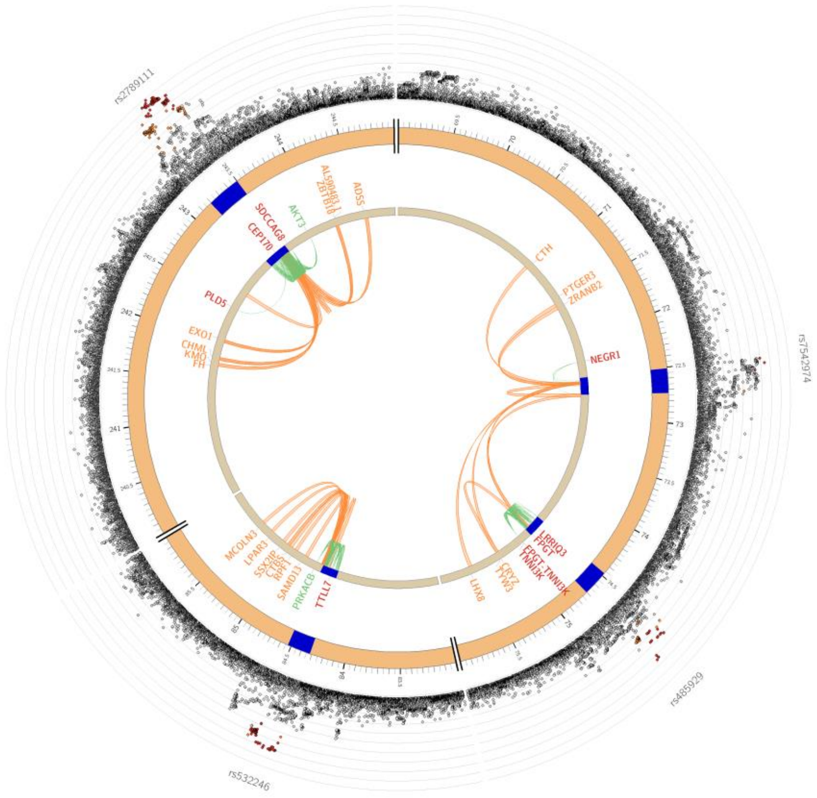

Figure S6b. Circos plot for MHQ data chromosome 2

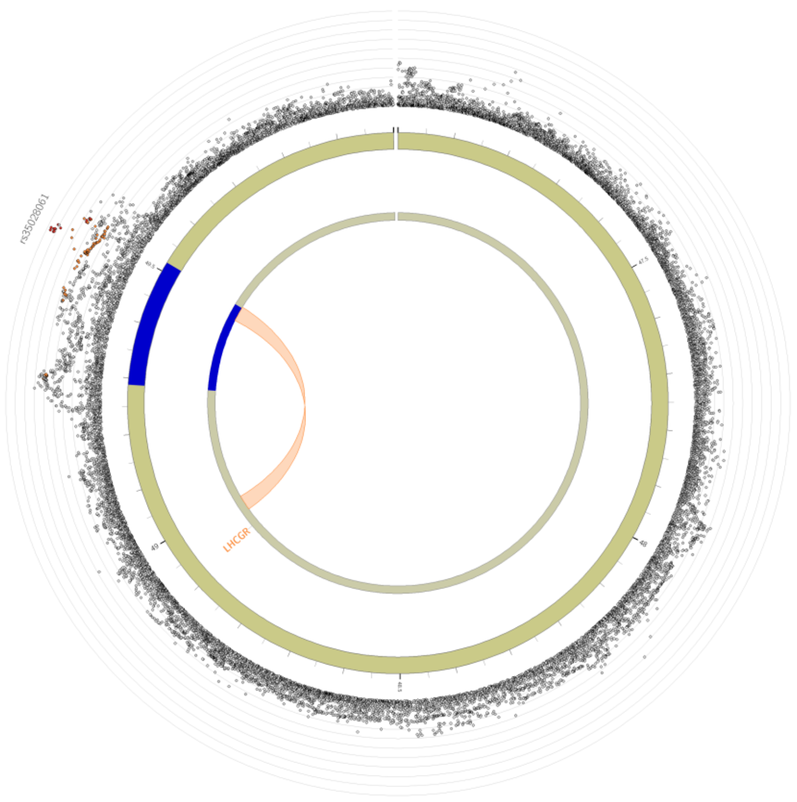

Figure S6c. Circos plot for MHQ data chromosome 3

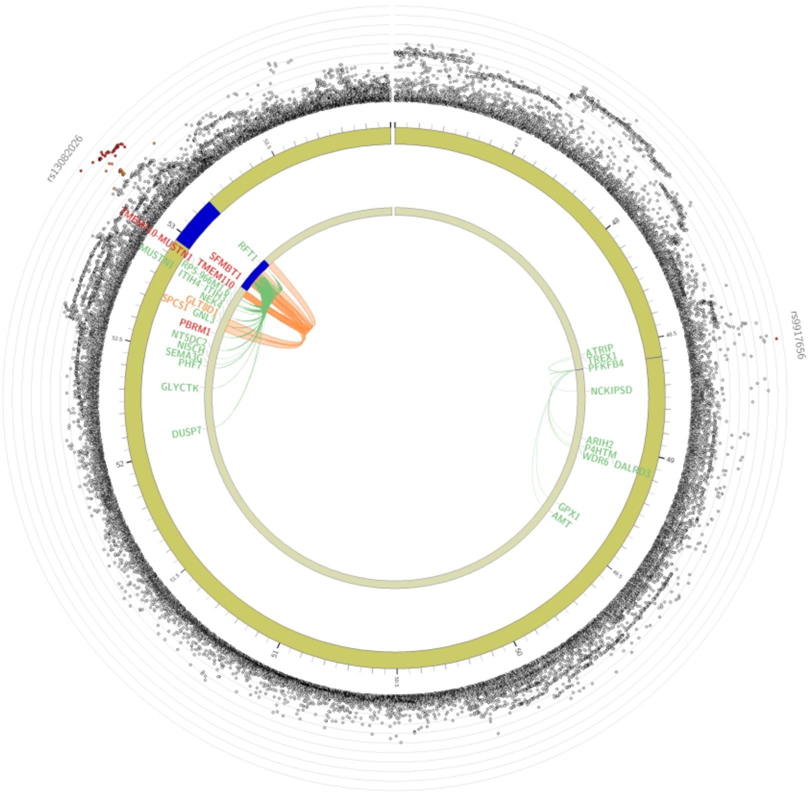

Figure S6d. Circos plot for MHQ data chromosome 4

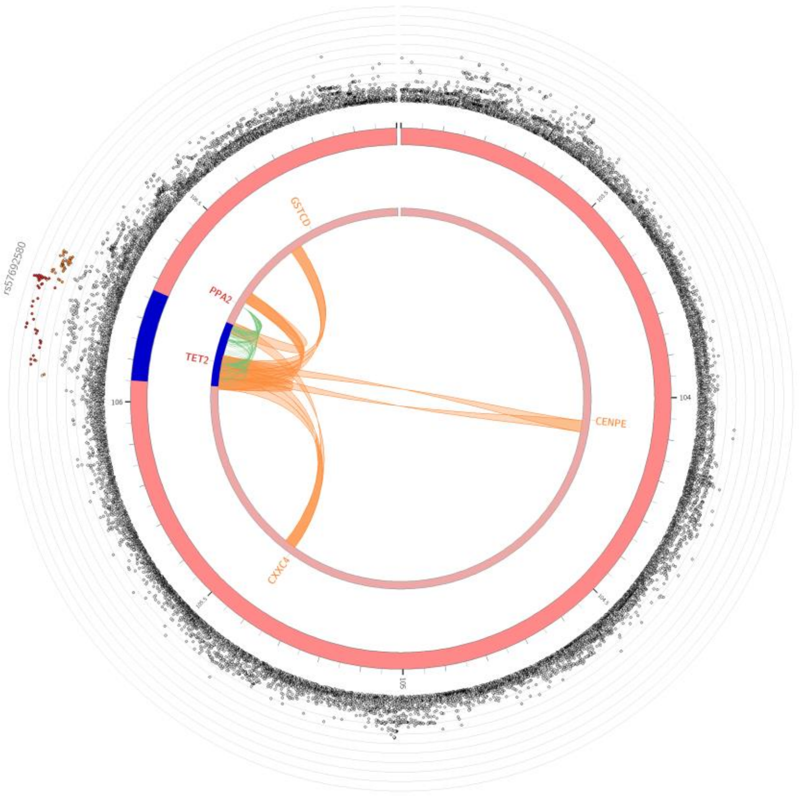

Figure S6e. Circos plot for MHQ data chromosome 5

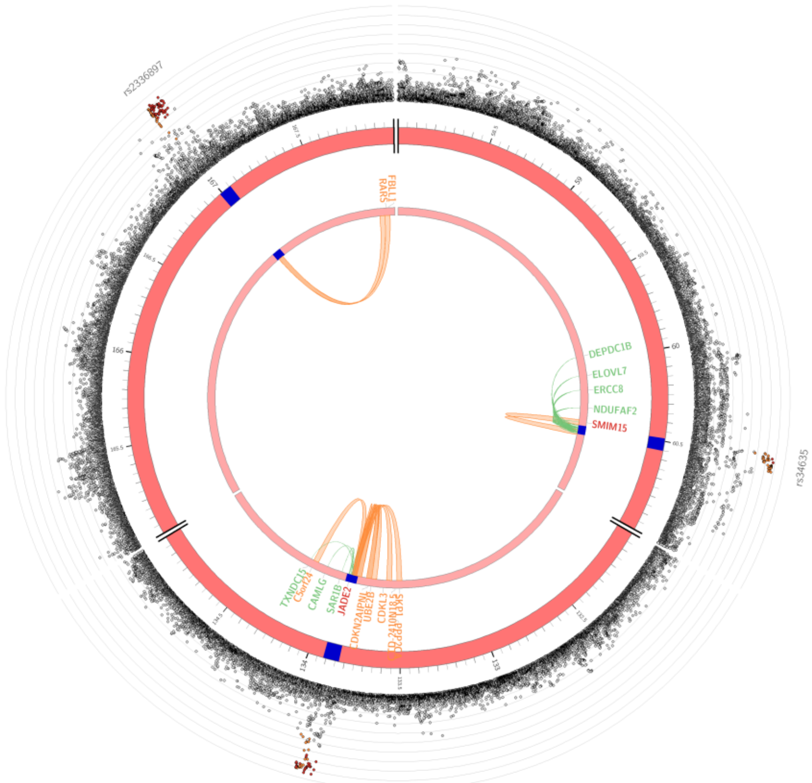

Figure S6f. Circos plot for MHQ data chromosome 6

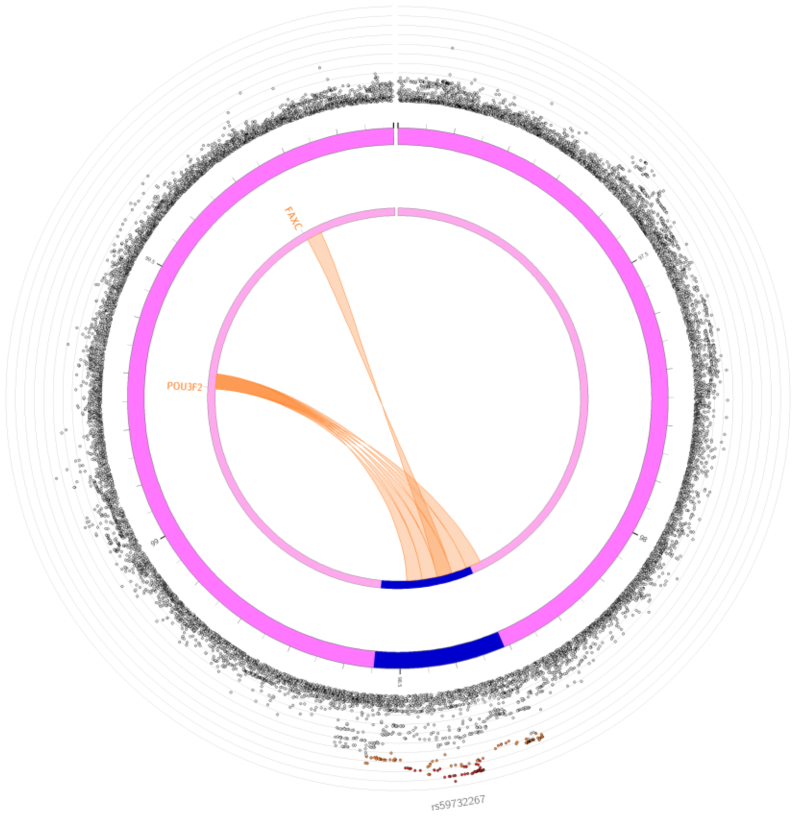

Figure S6g. Circos plot for MHQ data chromosome 8

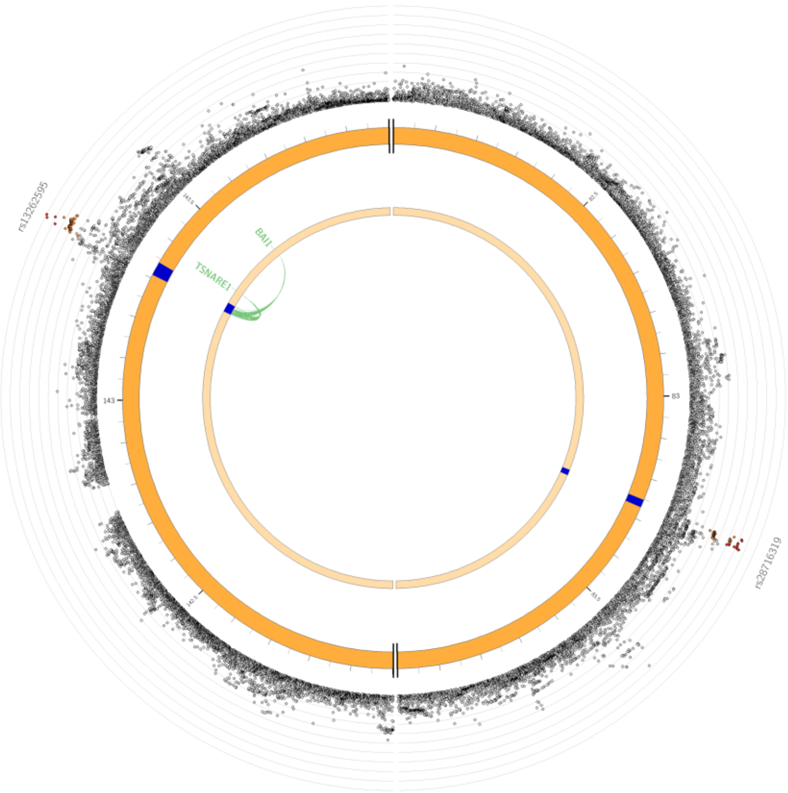

Figure S6h. Circos plot for MHQ data chromosome 9

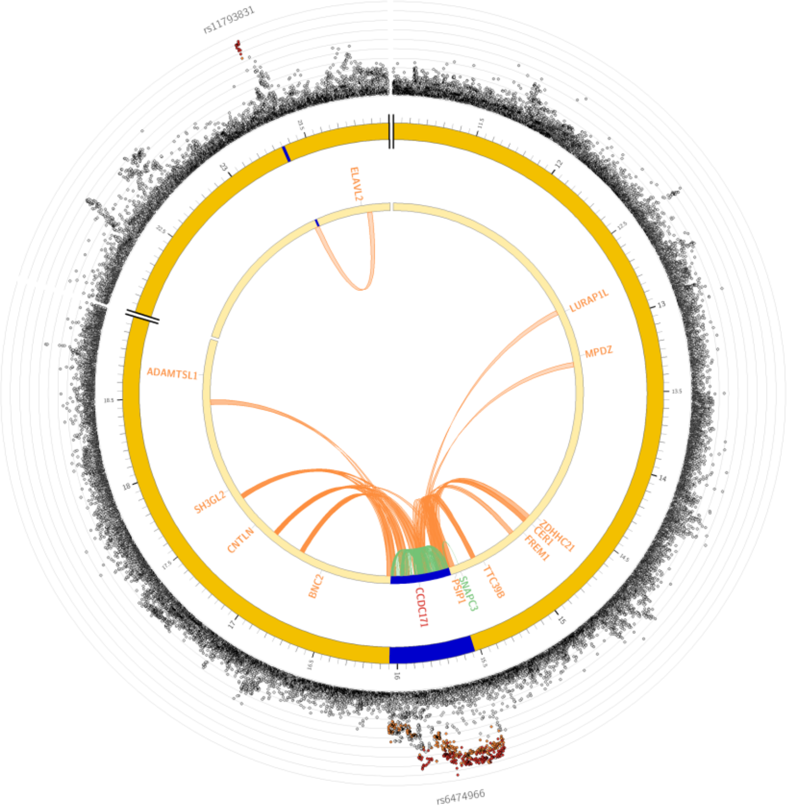

Figure S6i. Circos plot for MHQ data chromosome 11

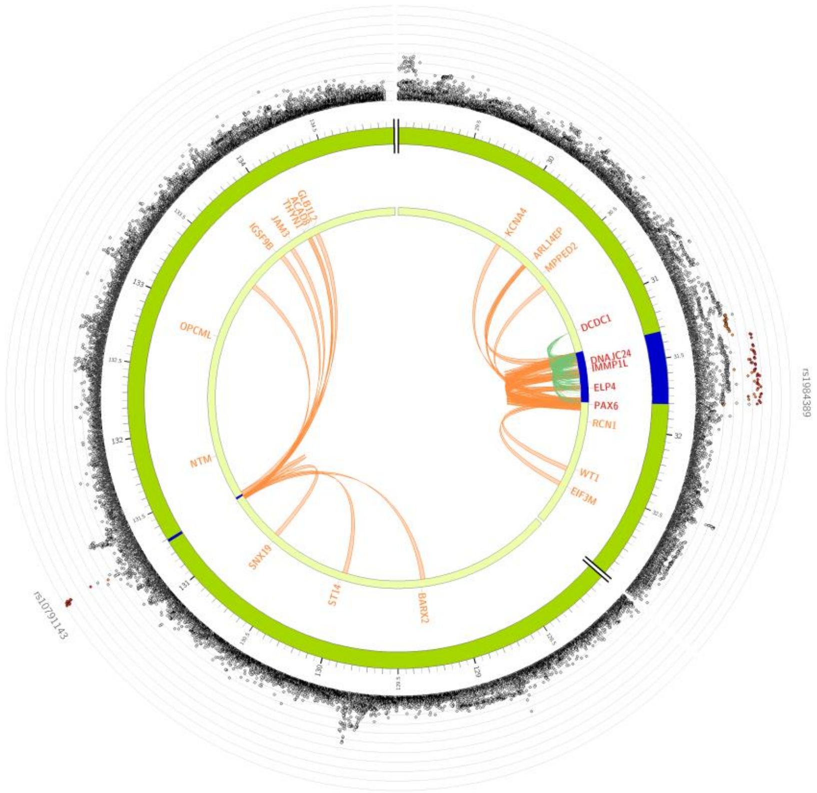

Figure S6j. Circos plot for MHQ data chromosome 16

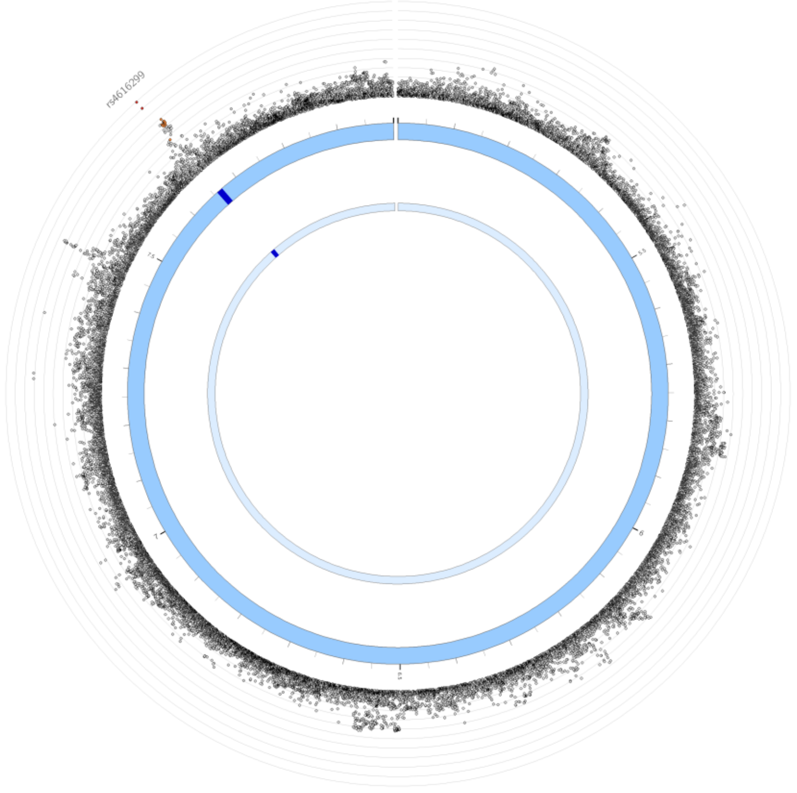

Figure S6k. Circos plot for MHQ data chromosome 17

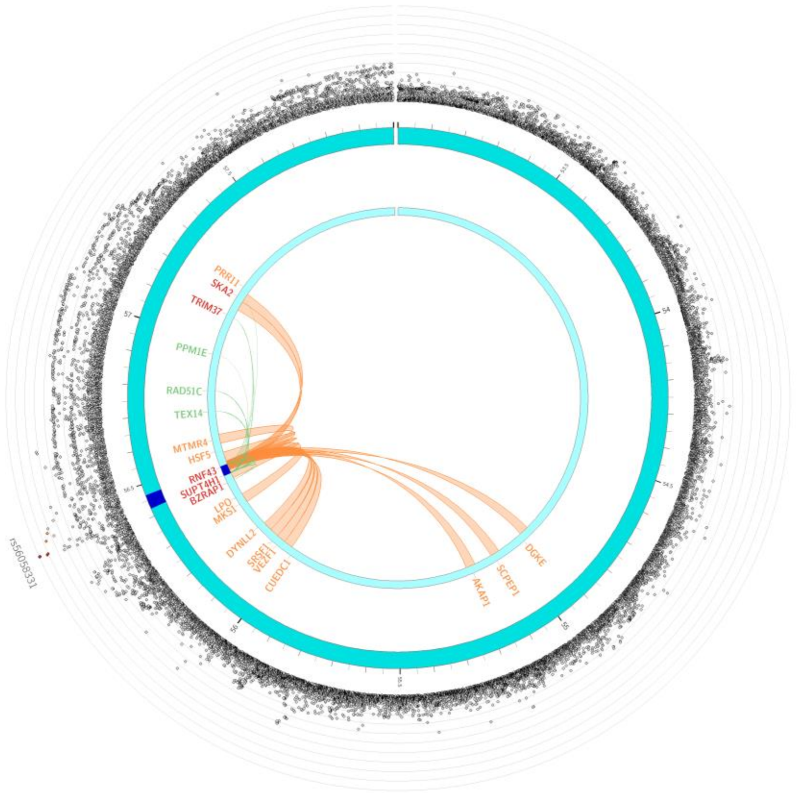

Figure S6l. Circos plot for MHQ data chromosome 18

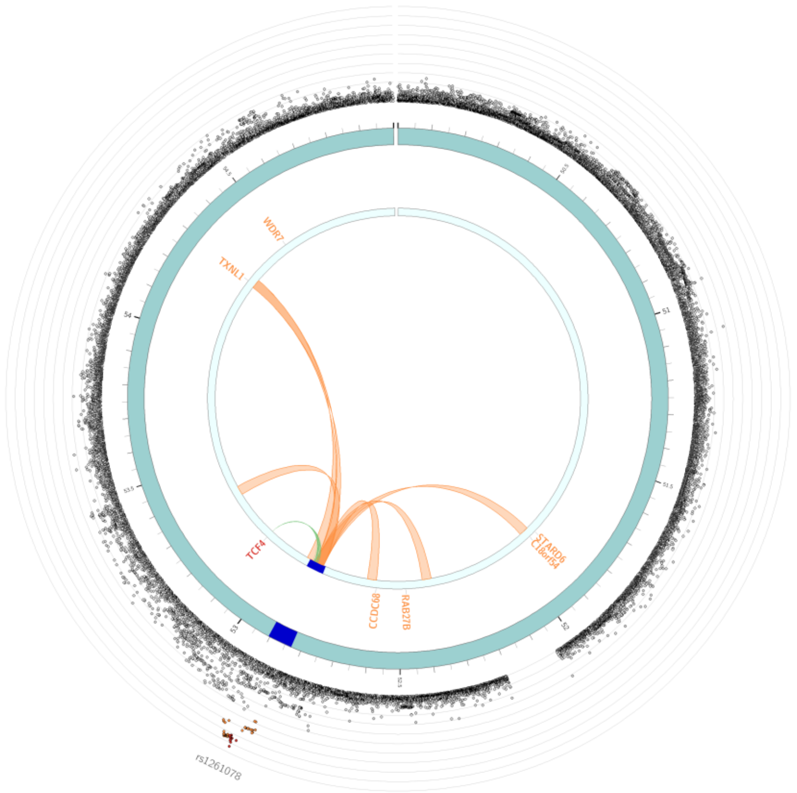

Figure S6m. Circos plot for MHQ data chromosome 19

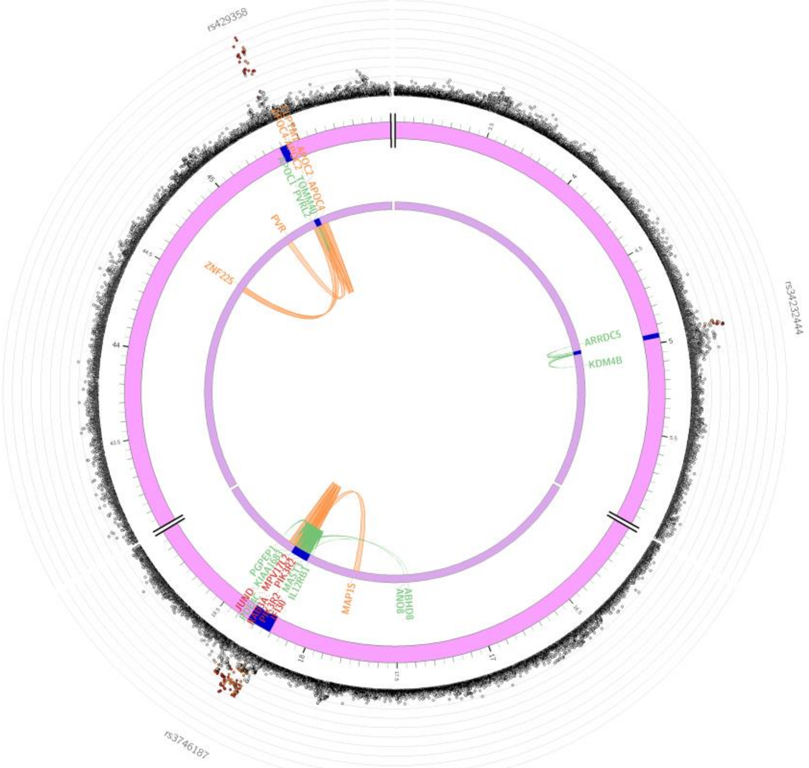

Figure S7. Comparison of effect sizes (as odds ratios) from top independent SNPs in the MHQ GWAS (x axis) compared to the MHQ GWAS whose deaths were reported before the recontact assessment period (y axis). Each SNP is plotted as overlaid with two sets of confidence intervals bars: 95% confidence interval (green) for assessing whether the effect size differs between the two GWAS; 99.999995% confidence interval (orange) for assessing whether the effect size differs from 1.0.

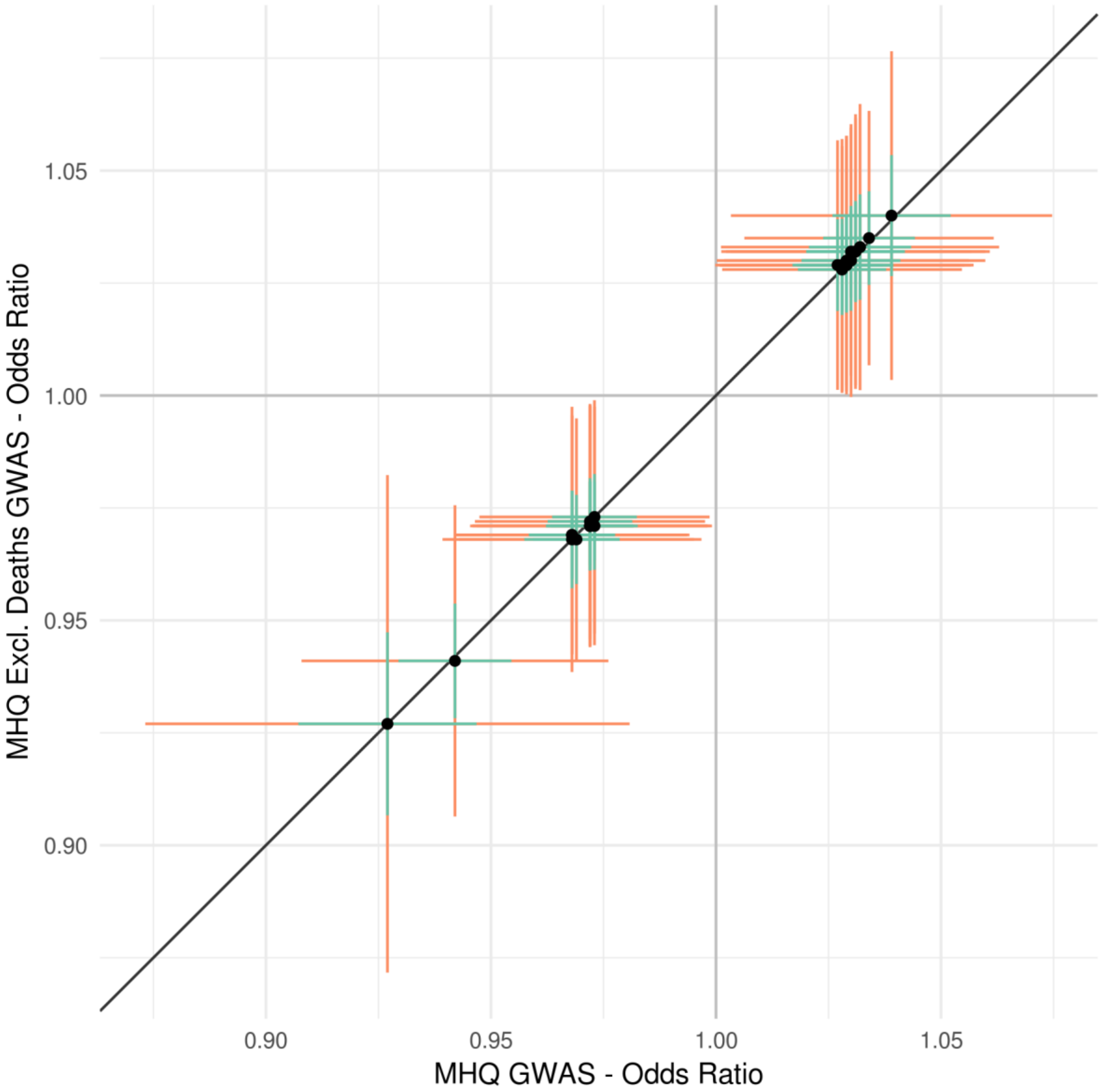

Figure S8. Filtering of the UK Biobank sample for genetic analysis. White British ancestry was determined by four-mean clustering of genetic principal components. Study overlap used genotype checksums to check for overlap with Psychiatric Genomics Consortium Major Depressive Disorder and Generation Scotland cohorts.

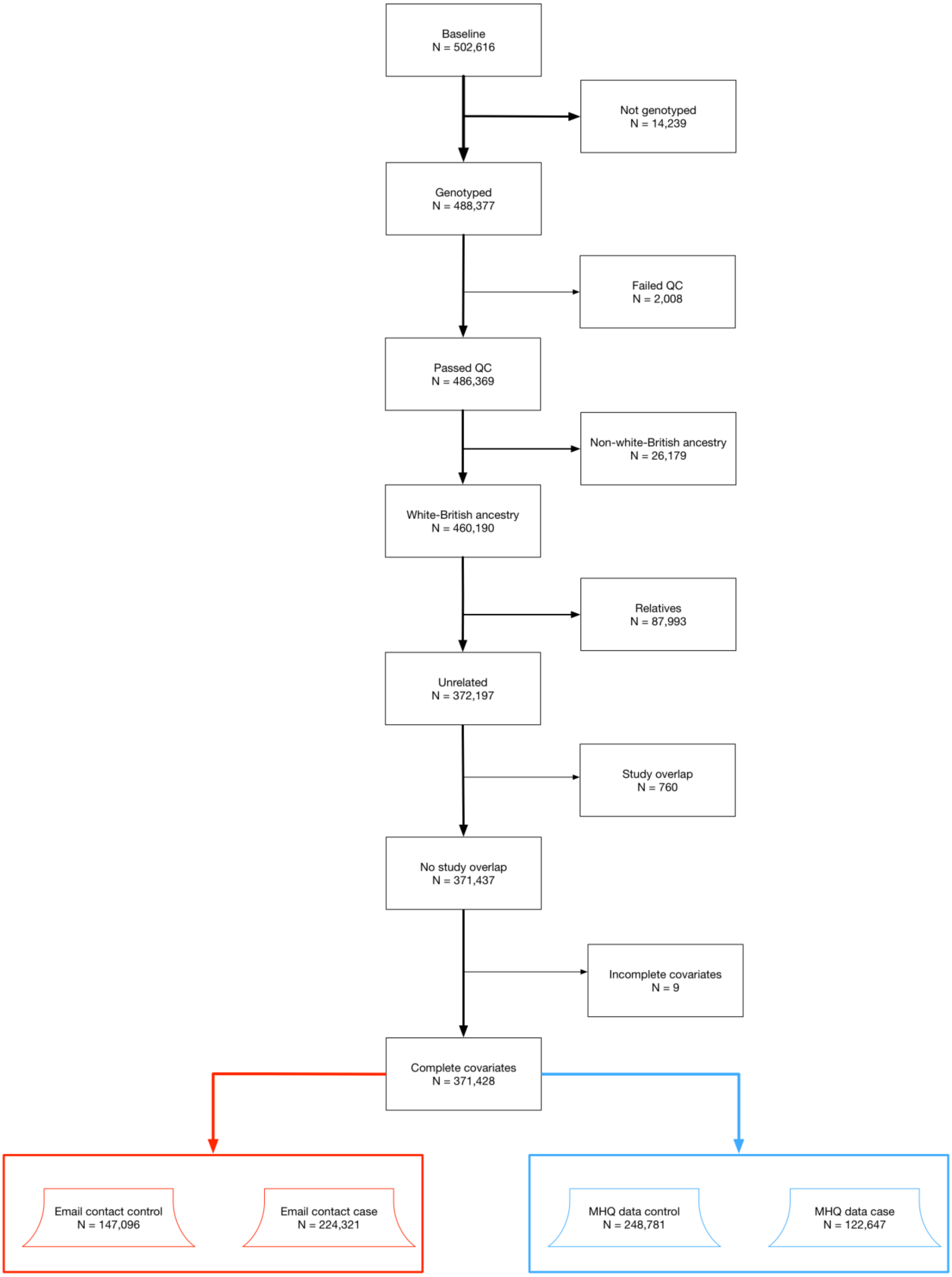

Supplement: dyz134_Supplementary_Data [file dyz134_supplementary_data.zip › dyz134-suppl_data/ije-2018-12-1551-File011.pdf]
